# Yeast applied readthrough inducing system (YARIS): an *invivo* assay for the comprehensive study of translational readthrough

**DOI:** 10.1093/nar/gkz346

**Published:** 2019-05-09

**Authors:** Petra Beznosková, Zuzana Pavlíková, Jakub Zeman, Colin Echeverría Aitken, Leoš S Valášek

**Affiliations:** 1 Laboratory of Regulation of Gene Expression, Institute of Microbiology ASCR, Videnska 1083, 142 20 Prague, the Czech Republic; 2 Biology Department and Biochemistry Program, Vassar College, 124 Raymond Avenue, Poughkeepsie 12601, NY, USA

## Abstract

Stop codon readthrough—the decoding of a stop codon by a near-cognate tRNA—is employed by viruses to balance levels of enzymatic and structural proteins and by eukaryotic cells to enable isoform-specific protein synthesis in response to external stimuli. Owing to the prevalence of premature termination codons in human disease, readthrough has emerged as an attractive therapeutic target. A growing list of various features, for example the +4 nucleotide immediately following the stop codon, modulate readthrough levels, underscoring the need for systematic investigation of readthrough. Here, we identified and described a complete group of yeast tRNAs that induce readthrough in the stop-codon tetranucleotide manner when overexpressed, designated readthrough-inducing tRNAs (rti-tRNAs). These rti-tRNAs are the keystones of YARIS (yeast applied readthrough inducing system), a reporter-based assay enabling simultaneous detection of readthrough levels at all twelve stop-codon tetranucleotides and as a function of the complete set of rti-tRNAs. We demonstrate the utility of YARIS for systematic study of translation readthrough by employing it to interrogate the effects of natural rti-tRNA modifications, as well as various readthrough-inducing drugs (RTIDs). This analysis identified a variety of genetic interactions demonstrating the power of YARIS to characterize existing and identify novel RTIDs.

## INTRODUCTION

Whereas the stop codons UAA, UAG and UGA are typically recognized in the ribosomal A-site by release factors (eRF1 and eRF3 in eukaryotes), competition by elongator tRNAs can infrequently result in accommodation of near-cognate tRNA, readthrough of the stop codon, and production of C-terminally extended protein isoforms (1). While stop codon readthrough is quite rare, occurring naturally at frequencies <0.1%, readthrough nonetheless plays important biological roles in cells: programmed readthrough has been observed to occur with frequencies up to 20% (2,3).

Both viruses and, more recently, eukaryotic cells have been shown to promote readthrough to modulate protein diversity. In viruses, programmed readthrough enables the production of a diverse array of structural and enzymatic proteins at appropriate stoichiometric levels (4,5). Similarly, eukaryotic cells employ programmed readthrough to product C-terminally extended protein isoforms with distinct functions (3,6,7). In one example, human cells employ programmed readthrough to produce strictly defined levels of enzymes whose additional C-terminal sequence targets them to the peroxisome (2,8). In the particular case of malate and lactate dehydrogenase, this targeting enables the establishment of a malate/lactate shuttle across the peroxisomal membrane.

Readthrough is also linked to disease. For mRNAs containing a premature stop codon (PTC), the frequency of readthrough defines the efficiency of functional protein synthesis (9). More than 15% of all human genetic diseases can be attributed to the presence of a PTC in the coding region of an essential protein (10). This fact has focused significant energy on the application and development of readthrough-inducing drugs (RTIDs), such as aminoglycoside antibiotics targeted at the ribosomal decoding center (11). However, likely because aminoglycosides do not target termination specifically but instead interfere with decoding generally, they are often toxic and result in variable patient outcomes (12). The design of more efficient RTIDs will require a more complete molecular understanding of both programmed and natural readthrough.

Because readthrough requires accommodation of a near-cognate elongator tRNA in the A-site, its efficiency depends on the degree to which this near-cognate tRNA competes against eRF1 for A-site binding and peptide bond formation. Previous work has demonstrated that the identity of the stop codon can affect the efficiency of readthrough. UGA is the leakiest of the stop codons, followed by UAG, and UAA is the most strictly recognized (13–15). In addition, the identity of the last two amino acids incorporated into the polypeptide chain (16) and the P-site tRNA also appear to modulate the efficiency of readthrough (17). Consistent with the emerging role of sequence context in all decoding events by the ribosome (18), the presence of stimulatory elements downstream of the stop codon also appears to influence the efficiency of readthrough (19–22), as does the identity of the +4 nucleotide immediately following the stop codon (23–26).

Until recently, however, the role played by the tRNA itself was not the central focus of inquiry. Nonetheless, natural variations in the relative abundance of distinct tRNAs have been observed among various yeast strains (27), as have strain-dependent differences in the levels of readthrough efficiency (1). In fact, two recent studies suggest that specific stop codons may be more susceptible to readthrough by specific near-cognate tRNAs (28,29): readthrough at UAA is most efficient with near-cognate tRNA^Tyr^ and tRNA^Gln^, whereas readthrough at UAG is most efficient with near-cognate tRNA^Tyr^ and UGA is most susceptible to readthrough by near-cognate tRNA^Trp^ and tRNA^Cys^. While readthrough was also observed in the presence of other near-cognate tRNAs—tRNA^Lys^ and tRNA^Arg^ at UAA and UGA and tRNA^Gln^, tRNA^Lys^ and tRNA^Arg^ at UAG— the efficiency of these events was markedly lower. The molecular determinants of these preferences remain mysterious.

We recently demonstrated that the stop codon, together with the +4 nucleotide, define a stop tetranucleotide that largely determines the observed preferences for specific near-cognate tRNAs (30–32). Analysis of readthrough at UGA—the leakiest of the three stop codons— revealed that, whereas the UGA-A tetranucleotide is preferentially readthrough by near-cognate tRNA^Trp^, the UGA-C and UGA-G tetranucleotides are readthrough most efficiently by near-cognate tRNA^Cys^ (30). Beyond determining near-cognate tRNA preference, the +4 nucleotide also appears to play a role in controlling the efficiency of readthrough: the presence of a cytosine at the +4 position modestly, but significantly, interferes with eRF1 decoding of all three stop codons (30), which is consistent with previous observations that readthrough at all stop codons with cytosine at the +4 position is the highest (1).

Intriguingly, we were able to identify a similar role for eukaryotic initiation factor 3 (eIF3) (31,33,34) in stimulating readthrough by near-cognate tRNAs with mismatches at the third, or wobble, position. eIF3 appears to interfere with eRF1 recognition of stop codons at the wobble position. Increased levels of improper eRF1•eRF3•GTP accommodation would enable greater competition of near-cognate tRNA with wobble mismatches and thus result in higher levels of readthrough. Together with the factors described above, the stop codon tetranucleotide and eIF3 contribute to an array of molecular determinants of readthrough efficiency. How the interplay of these factors is controlled to enable readthrough efficiencies from 0.1 to 20%, as observed in various cells and tissues under different conditions, remains unclear.

To enable systematic analysis of readthrough at multiple sequences in distinct genetic backgrounds and across different environmental conditions, we developed and present here the yeast applied readthrough inducing system (YARIS). We applied YARIS to monitor the efficiency of readthrough at the 12 distinct stop tetranucleotides, in the presence of elevated levels of all near-cognate tRNAs. Our results enabled us to define specificity rules for readthrough of specific stop tetranucleotides by specific near-cognate tRNA and characterize a new functional set of tRNAs responsible for these events: readthrough-inducing tRNAs (rti-tRNAs). In addition to tRNA^Trp^ and tRNA^Cys^ mentioned above, we show that tRNA^Tyr^ is an rti-tRNA for all four UAA-N tetranucleotides, as well as for the UAG-C. Whereas tRNA^Tyr^ prefers C at the +4 position of the UAG-N tetranucleotides, tRNA^Gln^*[tQ(CUG)M]* serves as an rti-tRNA at all four UAG-N tetranucleotides, although with a strong preference for UAG-G. We further employed YARIS to investigate the effect of specific tRNA modifications on the efficiency of readthrough induced by rti-tRNA. Specifically, we showed that: (i) Ψ35 of tyrosine tRNA is crucial for tyrosine incorporation at the UAG and UAA stop codons regardless of the identity of the +4 nucleotide; (ii) U34 of tRNA^Tyr^ is likely not a substrate for *PUS1* pseudouridine synthetase, as readthrough by this tRNA is unaffected in a PUS1 deletion strain; (iii) modification of tRNA^Tyr^ and tRNA^Cys^ by *MOD5* is important for their decoding at stop codons regardless of +4 nucleotide identify; and, (iv) methylation of C34 of tRNA^Trp^ interferes with its ability to promote tetranucleotide-specific readthrough. At last, we employed YARIS to interrogate how increased levels of specific rti-tRNA interact with the RTIDs G418, paromomycin, and tobramycin to affect readthrough at the complete set of stop tetranucleotides. This analysis reveals that, whereas certain stop tetranucleotides and their corresponding rti-tRNA are relatively insensitive to RTIDs, these molecules can specifically enhance readthrough by rti-tRNA at other stop tetranucleotides several-fold. These specific effects both illuminate gaps in our mechanistic understanding of RTIDs and may have implications for custom-tailored treatments targeted at inducing readthrough of specific PTCs.

## MATERIALS AND METHODS

### Yeast strains and plasmids

The lists and descriptions of plasmids and yeast strains used throughout this study (summarized in Supplementary Tables S1–3) can be found in the Supplementary Information.

### Stop codon readthrough assays and YARIS

The majority of stop codon readthrough assays in this study were performed using a standard bicistronic reporter construct bearing a *Renilla* luciferase gene followed by an in-frame firefly luciferase gene. Separating the two genes is either a tetranucleotide termination signal (UGA-C) or, for control purposes, the CAA sense codon followed by cytosine. In indicated cases, the termination signal and/or the following nucleotide context was modified. mRNA levels from reporters containing any stop codon tested do not differ from the CAA sense control (Supplementary Figure S1). It is noteworthy that this system avoids possible artifacts connected to the changes in the efficiency of translation initiation associated with the nonsense-mediated decay pathway (35), because both *Renilla* and firefly enzymes initiate translation from the same AUG codon. For further details please see (36). All experiments and data analyses were carried out according to the Microtiter plate-based dual luciferase protocol developed by (37) and commercially distributed by Promega. Readthrough values are represented as mean ± SD from triplicates (*n* = 6) and each experiment was repeated at least three times. The excel sheet for readthrough calculations with raw data of firefly and renilla measurements and with subsequent analyses of renilla and firefly levels for each reporter containing the stop codon tetranucleotide are given in the Supplementary Excel File (please see the panel ‘Renilla data’).

For YARIS, all readthrough values with their standard deviations are shown in the Supplementary Excel File, as well as their normalized values and the calculation of the log_2_ fold increase that was used to create the heatmaps using R. The scripts in R and the workflow are described in Supplementary Excel File (please see the panel ‘R script for heatmap’). In the corresponding assays, Tobramycin (AbCam;#ab120659); Paromomycine sulfate (Sigma;#110M1517) and Geneticin sulfate G418 (Gibco; #11811-031) were used.

### Northern blot analysis

The Quick RNA miniprep from yeast using glass beads for cell lysis was performed as previously described in (38). The RNAs were kept in RNAse free water, run on a Criterion™ Precast Gel 15% TBE-Urea, 1.0 mm (Bio-Rad) and transferred to the 0.45 nylon transfer membrane (Nytran SPC, Whatman). Custom made 5′ ^32^Plabeled oligonucleotides were used as probes. Signals were captured in Fuji MS phosphor storage screens, scanned with a Molecular Imager FX (Bio-Rad) and quantified with NIH ImageJ.

### RNA isolation, reverse transcription and qPCR

The Quick RNA miniprep from yeast cells using glass beads for cell lysis was performed as previously described in (38). After TurboDNAse digestion (Ambion, cat # AM2238), cDNA was synthesized using the high-capacity cDNA reverse transcription kit (Applied Biosystems, # 4368813). Quantitative polymerase chain reaction (qPCR) was performed using 5 × HOT FIREPol EvaGreen qPCR Mix Plus (Solis BioDyne # 08-25-00020). The obtained data were normalized to the reference 5S rRNA; at least two individual experiments were performed. qPCR primers are listed in Supplementary Table S3.

## RESULTS

### tRNA^Tyr^*[tY(GUA)J2]* and tRNA^Gln^*[tQ(CUG)M]* are the specific readthrough substrates for the UAA-N and UAG-N tetranucleotides in budding yeast

Using a dual-luciferase assay we have previously observed that only suppressor tRNA and tRNAs with mismatches at the wobble position can, when overexpressed, promote stop codon readthrough (Supplementary Figure S2A; gray area). In particular, we have shown that tRNA^Trp^ is the specific readthrough substrate for the UGA-A tetranucleotide *in vivo*, as compared to all other near-cognate and some non-cognate tRNAs that we tested (for the comprehensive list see Supplementary Figure S2), and tRNA^Cys^ is preferred at UGA-G, while at UGA-C and UGA-U both near-cognate tRNAs are incorporated (30). To complete this picture, we used this same approach to identify the specific readthrough substrates for the UAA-N and UAG-N tetranucleotide sets.

**Figure 1.**
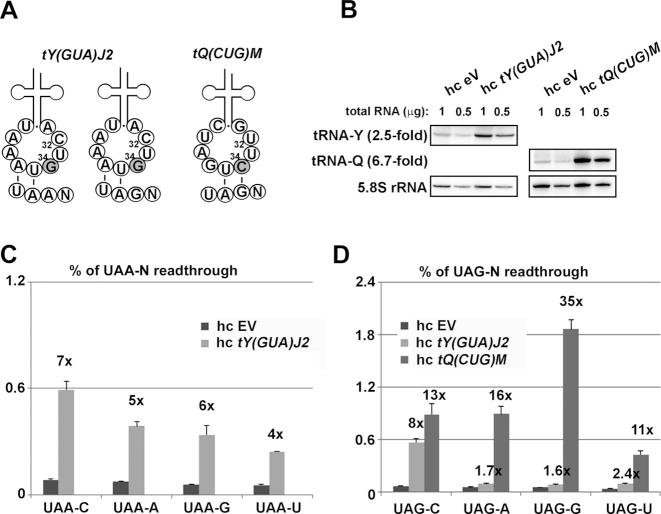
tRNA^Tyr^*[tY(GUA)J2]* and tRNA^Gln^*[tQ(CUG)M]* are the specific readthrough substrates for the UAA-N and UAG-N tetranucleotides in budding yeast. (**A**) Schematics of Tyr and Gln tRNAs base-pairing with the indicated stop codon tetranucleotides. Only the nucleotides of the anticodon loop are shown with the third stop codon base N_34_ (in gray) and N_32_ indicated. (**B**) Increased gene dosage of *tY(GUA)J2* or *tQ(CUG)M* tRNAs increases their cellular levels *in vivo*. Total RNAs were extracted from the PBH156 strain bearing a plasmid indicated at the top of each panel and 1 or 0.5 μg aliquots were loaded onto the Criterion Precast gels and subjected to northern blot with ^32^P-labeled probes shown on the left. (**C**) Increased gene dosage of *tY(GUA)J2* elevates readthrough levels on all four UAA-N tetranucleotides. The PBH156 was transformed with either empty vector (EV) or high copy (hc) tY(GUA)J2 and the resulting transformants were grown in SD and processed for stop codon readthrough measurements using standard dual luciferase readthrough reporter constructs YEp-R/T-CAAC-L; YEp-R/T-UAAC-L; PBB157; PBB161 and PBB162, as described in ‘Materials and Methods’ section. Readthrough values are represented as mean ± SD from quintuplicates (*n* = 5) and each experiment was repeated at least three times. (**D**) The impact of an increased gene dosage of *tY(GUA)J2* or *tQ(CUG)M* on the UAG-N readthrough. The PBH140 derivative was transformed with either EV, hc tY(GUA)J2 or hc tQ(CUG)M and the resulting transformants were grown in SD and processed for stop codon readthrough measurements using standard dual luciferase readthrough reporter constructs YEp-R/T-CAAC-L; YEp-R/T-UAGC-L; PBB159; PBB160 and PBB158, as described in ‘Materials and Methods’ section. Readthrough values are represented as mean ± SD from quintuplicates (*n* = 5) and each experiment was repeated at least three times.

While our previous work demonstrated that tRNA^Tyr^*[tY(GUA)J2]* is measurably incorporated at both UAA-C and UAG-C tetranucleotides (31), work by others has also shown that amino acids other than tyrosine can be incorporated into polypeptide chains upon readthrough of UAA and UAG stop codons (28,29). To account for this, we tested readthrough at the UAA-N and UAG-N tetranucleotides in the presence of elevated levels of near-cognate tRNAs possessing anticodon mismatches at either the third/wobble position (tRNA^Tyr^*[tY(GUA)J2]*—Figure 1A), or at the first position (tRNA^Gln^*[tQ(CUG)M]*—Figure 1A, and tRNA^Gln^*[tQ(UUG)]*, tRNA^Lys^*[tK(CUU)C]* and tRNA^Glu^*[tE(CUC)D]*—Supplementary Figures S3A and 4A). We are not aware of any evidence that near-cognate tRNAs with mismatches at the second position promote readthrough (1). Cellular levels of each near-cognate tRNA were elevated by the incorporation of hc plasmids carrying the corresponding gene. Elevated levels of each tRNA were confirmed via northern blot (Figure 1B; Supplementary Figures S3B and 4B).

Consistent with our previous observations, the wobble-mismatched tRNA^Tyr^*[tY(GUA)J2]* increased readthrough levels at both UAA-C and UAG-C tetranucleotides, 7- and 8-fold, respectively, as compared to readthrough levels observed in the presence of high-copy empty vector (EV). These effects are beyond the already 2-fold increase in readthrough efficiency caused by impaired eRF1 decoding when cytosine is present at the +4 position of the stop tetranucleotide (30). In fact, we observe that tRNA^Tyr^*[tY(GUA)J2]* is capable of inducing readthrough at all four UAA-N tetranucleotides, increasing readthrough levels 7-, 5-, 6- and 4-fold at the UAA-C, UAA-A, UAA-G and UAA-U tetranucleotides, respectively (Figure 1C), whereas the near cognate tRNA^Gln^*[tQ(UUG)B]* and tRNA^Gln^*[tQ(UUG)L]*, which contain mismatches at the first position, do not markedly affect readthrough at the UAA-N tetranucleotides (Supplementary Figure S3C). Interestingly, at UAG-N tetranucleotides, however, the ability of tRNA^Tyr^*[tY(GUA)J2]* to increase readthrough is restricted only to the UAG-C tetranucleotide (Figure 1D).

In contrast, the tRNA^Gln^*[tQ(CUG)M]*, which is cognate for the second and third nucleotides of the stop codon, was able to induce readthrough at all UAG-N tetranucleotides, increasing readthrough 13-, 16-, 35- and 11-fold at UAG-C, UAG-A, UAG-G and UAG-U, respectively (Figure 1D). This contrasts with the inability of the other tRNA^Gln^ isoacceptors—tRNA^Gln^*[tQ(UUG)B]* and tRNA^Gln^*[tQ(UUG)L]*—to promote readthrough at their corresponding near-cognate UAA-N tetranucleotides (Supplementary Figure S3C). Beyond their distinct anticodons, there are only minor sequence variations across these three isoacceptors (Supplementary Figure S5). The origin of these dramatic readthrough differences is an attractive target for future inquiry. Of the remaining near-cognate tRNAs containing mismatches in the first position we tested, neither tRNA^Lys^*[tK(CUU)C]* nor tRNA^Glu^*[tE(CUC)D]* induced increased readthrough at the UAG-N tetranucleotides (Supplementary Figure S4), which is consistent with structural studies from bacteria where U-U and C-U base pairing is not tolerated at the first position of the codon-anticodon helix (39,40).

**Figure 2.**
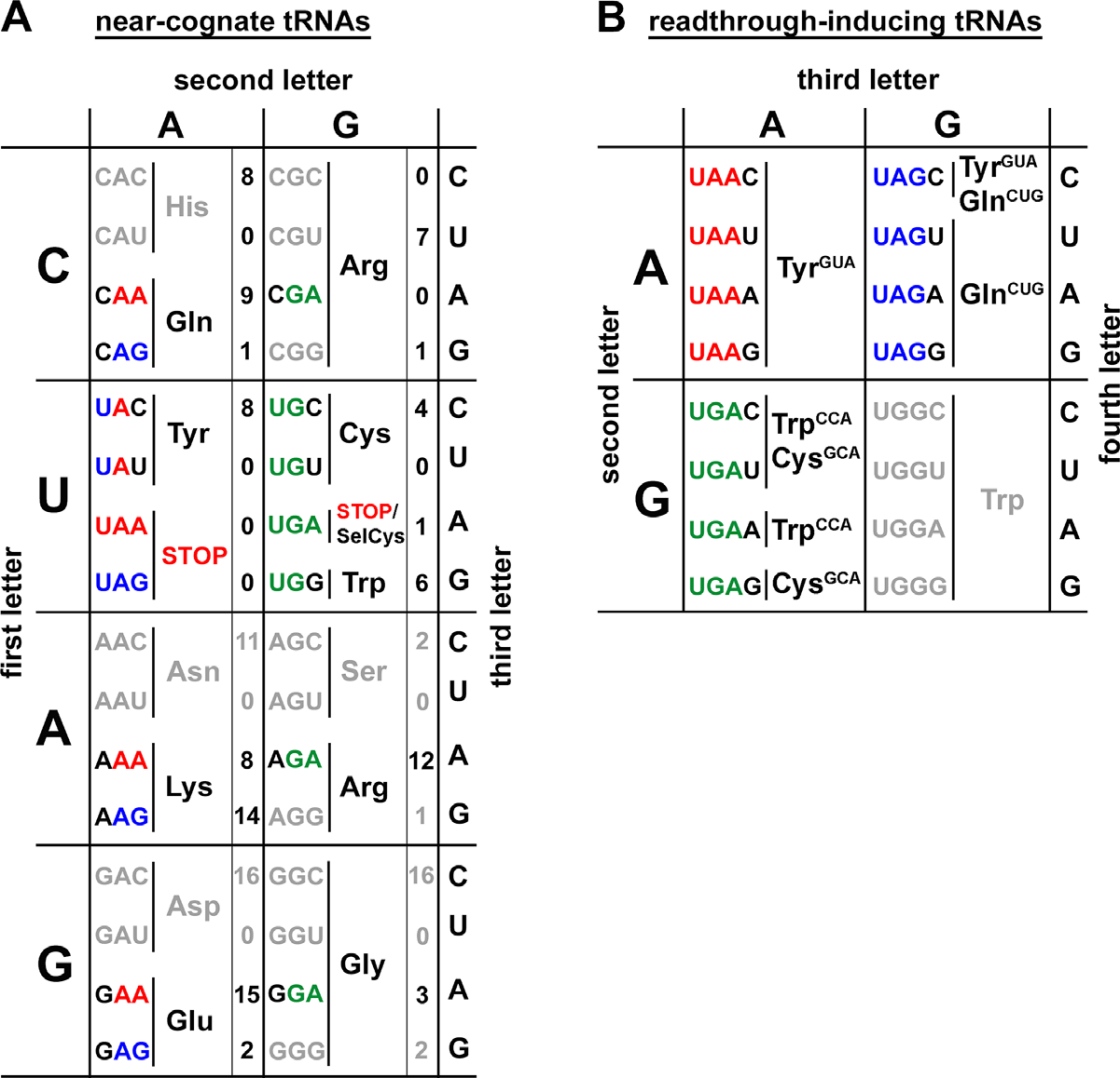
Expanded decoding rules defining readthrough inducing tRNAs (rti-tRNAs) from the pool of all near-cognate tRNAs. (**A**) A modified schematic of the genetic code to highlight all existing near-cognate tRNAs for all three stop codons; numbers of genes coding for individual near-cognate tRNAs occurring in the entire *Saccharomyces cerevisiae* genome are given (source SGD). (**B**) A schematic illustrating the expanded decoding rules for specific incorporation of individual rti-tRNAs at individual stop codons determined by the nucleotide immediately following the stop codon (+4; fourth letter).

Together with our previous work, our analyses of more than 33 anticodon-stop codon combinations (Supplementary Figure S2) outline a potentially complete set of tRNAs capable of inducing efficient readthrough at the various stop-codon tetranucleotides, a class of tRNAs we dub readthrough-inducing tRNAs (rti-tRNA). These represent a subset of those tRNAs whose anticodon is near-cognate (here defined as one mismatch at either the first or third position) (Figure 2A), with specificity conferred by the full stop-codon tetranucleotide (Figure 2B). (Indeed, we cannot rule out the possibility that exceptions to these general rules may occur under specific conditions). Please note that for rti-tRNAs there are no isodecoders with a different primary sequence in the *Saccharomyces cerevisiae* genome (Supplementary Figure S5A for CUG anticodon; and Supplementary Figure S6 for CCA, GCA and GUA anticodons).

### Systematic definition of stop-codon tetranucleotide decoding specificity by rti-tRNAs using the yeast applied readthrough inducing system (YARIS)

**Figure 3.**
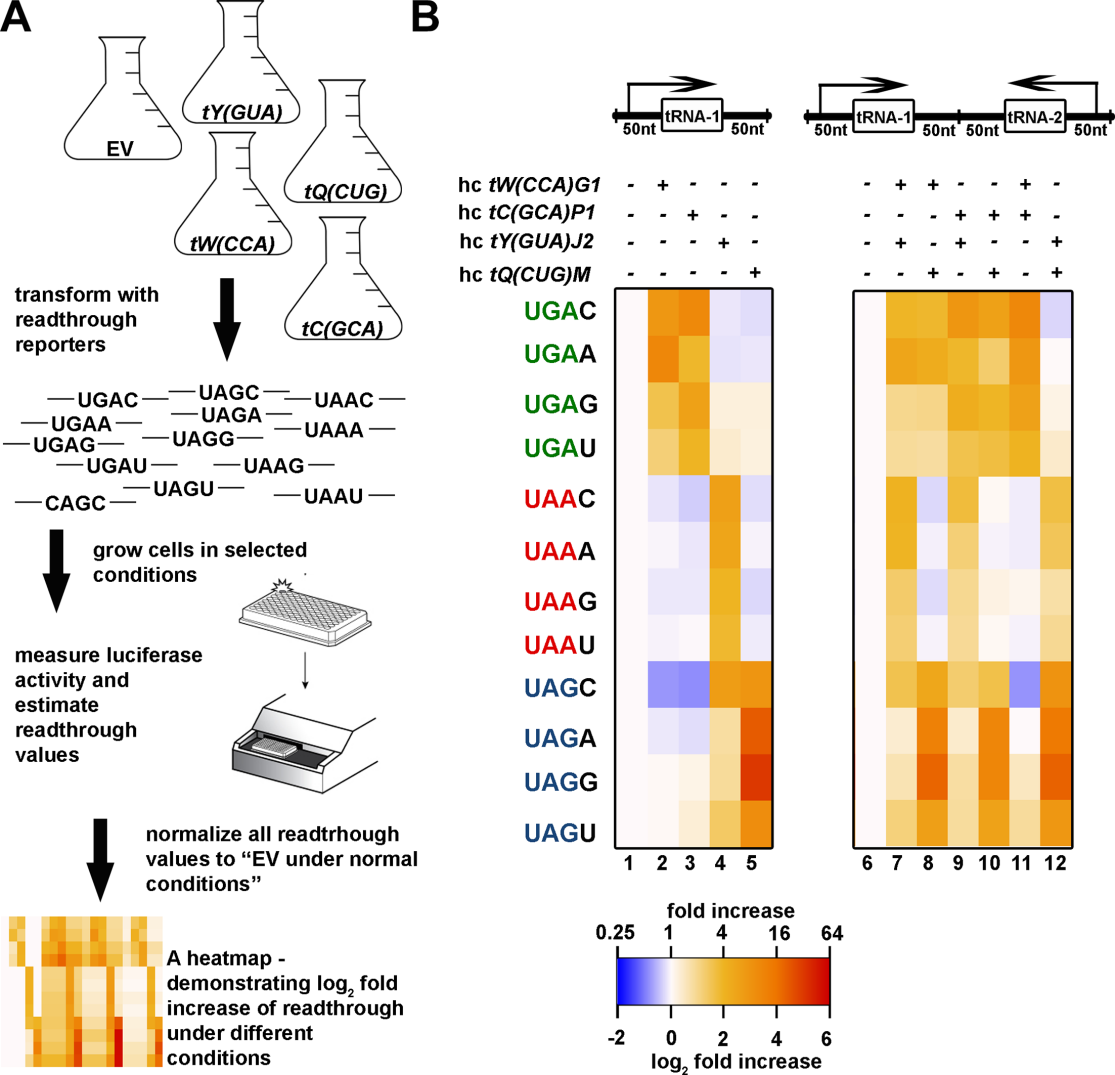
YARIS. (**A**) An experimental outline of YARIS. Yeast cells of interest are transformed with two plasmids; the first plasmid bears an rti-tRNA of interest or an EV, while the second one carries a dual luciferase readthrough reporter with either varying stop codon tetranucleotides or a control sense codon (CAG). Cells are grown under selected conditions and lysates are prepared and subjected to measurements of luciferase activity according to the vendor. Readthrough values are normalized to cells bearing the EV and the log_2_ fold increases in readthrough are expressed as heatmaps created using R. (**B**) The Log2 fold increase heatmap of the readthrough induction in the PBH156 strain expressing various combinations of rti-tRNAs (columns) with stop codon tetranucleotides (rows).

While our previous work delineated a set of rti-tRNA that promote readthrough at specific stop-codon tetranucleotides (30,31), we were not able to exhaustively test the complete matrix of stop codon tetranucleotides and rti-tRNAs. To enable such a systematic analysis, we developed the YARIS. YARIS expands upon our previous dual-luciferase experiments, enabling the comparison of readthrough levels observed in the presence of high-copy plasmids expressing rti-tRNAs versus control EVs on reporter constructs for all 12 stop codon tetranucleotides. This approach can be further expanded to test these effects across various conditions (Figure 3A), such as the presence or absence of RTIDs. In total, 60 unique combinations are tested in each individual experiment. This matrix of effects can be visualized as a heatmap of log_2_-fold increases in readthrough, as compared to the empty-vector control (Figure 3B). The values of each tile in the heatmap are derived from three independent measurements.

We first asked how the four rti-tRNAs we have previously described affect readthrough across the full complement of stop-codon tetranucleotides in the WT (PBH156) used in our previous studies (30,31). The resulting heatmap highlights the specificity of each rti-tRNA for its corresponding stop-codon tetranucleotide (Figure 3B, left panel). (Calculated means, fold-changes and associated errors for each tile are shown in the Supplementary Excel File.) Elevated levels (as confirmed by northern blot, see Figure 1B) of tRNA^Gln^*[tQ(CUG)M]* result in increased readthrough at the UAG-N set of stop-codon tetranucleotides (Figure 3B, left panel column 5), whereas the UAA-N and UGA-N tetranucleotides are largely unaffected. Notably, the hierarchy of effects observed in the presence of the various +4 nucleotides is similar to those observed in our previous experiments, in particular the dramatic increase observed at the UAG-G tetranucleotide. Similarly, elevated levels of tRNA^Tyr^*[tY(GUA)J2]* specifically increase readthrough at UAA-N set of tetranucleotides and UAG-C (Figure 3B, left panel column 4), and elevated levels of either tRNA^Cys^*[tC(GCA)P1]* or tRNA^Trp^*[tW(CCA)G1]* exclusively increase readthrough at the UGA-N tetranucleotide set (Figure 3B, left panel columns 2 and 3).

Having demonstrated the utility of YARIS by recapitulating the results of several of our previous studies in one experiment and showing that three stop codon tetranucleotides have not one but two rti-tRNAs (Figure 2B), we next asked how the simultaneous overexpression of these two rti-tRNAs from a single plasmid would affect readthrough at their target stop tetranucleotides. To this end we employed YARIS to follow all possible tRNA and stop tetranucleotide combinations (Figure 3B, right panel), with a special focus put on tRNA^Trp^*[tW(CCA)G1]* in combination with tRNA^Cys^*[tC(GCA)P1]* at UGA-C or UGA-U, and tRNA^Tyr^*[tY(GUA)J2]* combined with tRNA^Gln^*[tQ(CUG)M]* at UAG-C. Dual overexpression of these tRNAs from one plasmid did not produce the same levels of readthrough we observed in the presence of individual overexpressed rti-tRNA. Consistently, when we analyzed the tRNA levels by northern blot, we observed that tRNA levels in dual tRNA overexpressions are also lower than a single tRNA overexpression (Supplementary Figure S7). While this effect complicates the analysis of additive effects as compared to individual rti-tRNA overexpression, the relative additivity of tRNA pairs can be inferred by comparison within this set. The combination of tRNA^Trp^*[tW(CCA)G1]* and tRNA^Cys^*[tC(GCA)P1]* in column 11 causes significantly higher induction at all UGA tetranucleotides, not only at UGA-C and UGA-U (Figure 3B; compare with columns 7 and 8 for tryptophan in combination with two other rti-tRNAs with different specificity; and with columns 9 and 10 for cysteine in combination with rti-tRNAs with different specificity). This suggests that both near-cognate tRNAs can incorporate at UGA codons, with the level of incorporation depending on the +4 nucleotide and the decoding rule we have described here (summarized in Figure 2B). The combination of tRNA^Tyr^*[tY(GUA)J2]* and tRNA^Gln^*[tQ(CUG)M]* results in a higher induction only at the UAG-C tetranucleotide, confirming that tyrosine incorporation contributes to readthrough at UAG only when followed by C. The remaining four combinations (column 7–10) follow the established decoding rules and patterns observed in the presence of individual rti-tRNA overexpression (Figure 3B, left panel). A global comparison of the patterns of readthrough efficiency observed in the presence of either individual or pairwise rti-tRNA overexpression confirms that the presence of multiple rti-tRNA expands the set of stop tetranucleotides at which readthrough occurs. This comparison further reveals that UGA, and to a lesser extent UAG, are more leaky when two rti-tRNAs are overexpressed. All together, these effects are consistent with the high-specificity of each rti-tRNA for its corresponding stop-codon tetranucleotide set and further underscore the complex portfolio of phenotypes that might be observed in the presences of multiple rti-tRNA.

### Dissection of the readthrough effects of distinct tRNA modifications using YARIS

We next employed YARIS to investigate the effects of distinct tRNA modifications on the efficiency of readthrough in the presence of elevated levels of individual rti-tRNA at each of the 12 stop tetranucleotides. To this end, we monitored readthrough levels in deletion strains for several tRNA modifying enzymes responsible for modifications occurring in the rti-tRNA (Supplementary Figure S8) and compared these to readthrough levels observed in the corresponding wild-type (WT) strain. Deletion and matching WT strains were obtained from the Saccharomyces Genome Deletion Project. Genomic deletions were verified by PCR prior to use. We confirmed the elevated levels of all rti-tRNAs upon overexpression in each strain by northern blot analysis (Supplementary Figure S9).

PUS1 is a general RNA pseudouridine synthase which introduces pseudouridine modifications at positions 26–28, 34–36, 65 and 67 on tRNA substrates (41). Of all rti-tRNAs, this modification affects tRNA^Trp^*[tW(CCA)G1]*, which contains pseudouridine at positions 26–28 and 65, as well as tRNA^Tyr^*[tY(GUA)J2]*, which contains a pseudouridine at the second position of the anticodon (U35). This latter modification is also performed by PUS7 (41) and results in stronger base pairing than that of a canonical A:U pair, perhaps by rigidifying the sugar-phosphate backbone and forcing the Ψ:A pair to adopt the A-form conformation observed in a genuine Watson–Crick pair, resulting in improved stacking over the neighboring base pair (42–44). Previous work has shown that in a *pus7Δ* strain, tRNA^Tyr^*[tY(GUA)J2]* lacking Ψ35 is less efficiently incorporated at both UAA and UAG stop codons, with this effect being greater at UAA (44). Notably, global readthrough at UAA was not affected—despite the decreased incorporation of tRNA^Tyr^*[tY(GUA)J2]* lacking Ψ35—because tRNA^Gln^*[tQ(UUG)]* replaced the role of tRNA^Tyr^*[tY(GUA)J2]* in this background.

**Figure 4.**
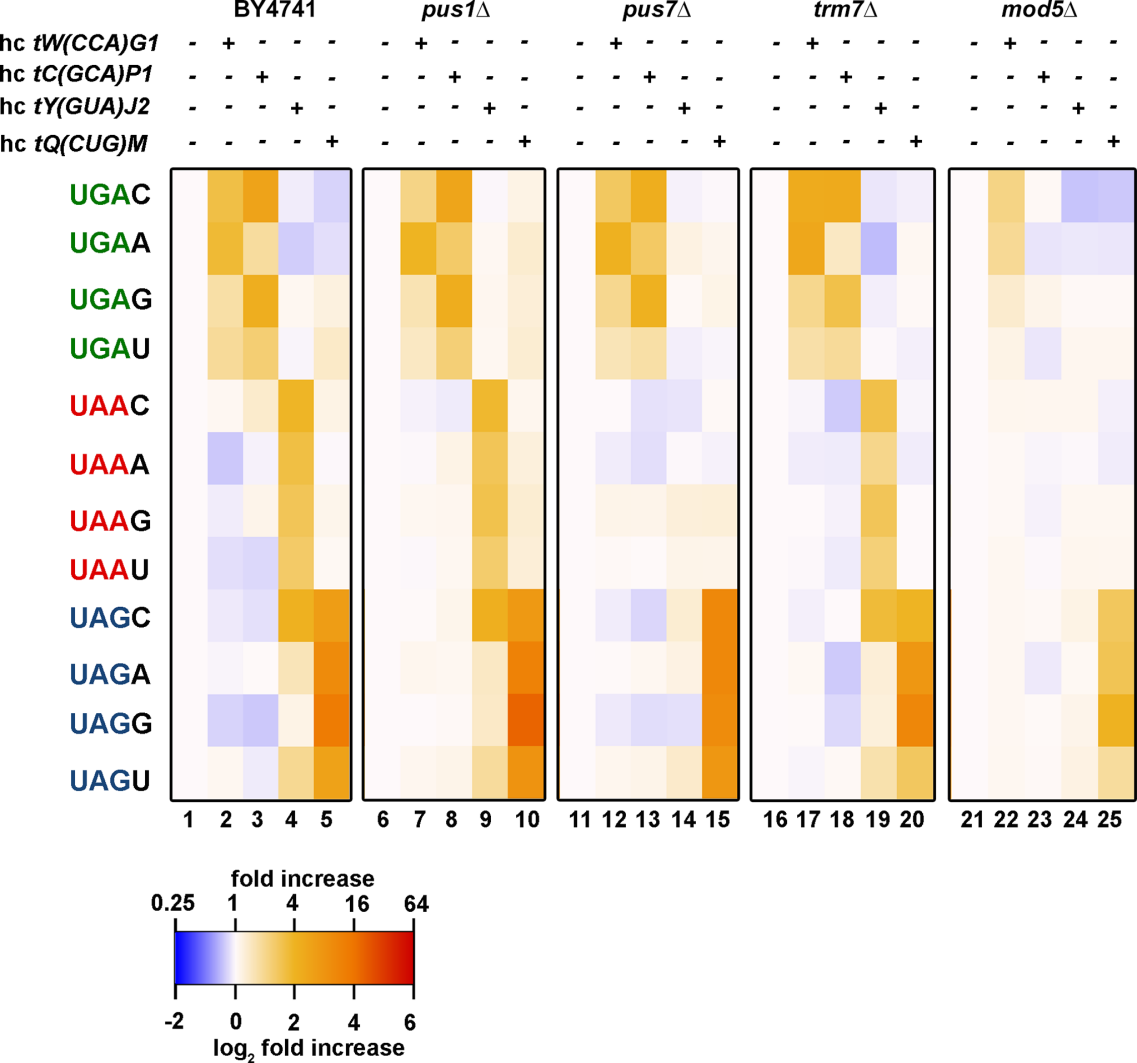
Dissection of the readthrough effects of distinct tRNA modifications using YARIS. The Log2 fold increase heatmap of the readthrough induction in strains individually deleted for genes encoding selected tRNA modifying enzymes expressing various combinations of rti-tRNAs (columns) with stop codon tetranucleotides (rows).

To explore the effects of these modifications more systematically, we subjected the *pus1Δ* and *pus7Δ* strains to YARIS. In the *pus1Δ* background, we observe little to no effect on readthrough as compared to the WT background, where readthrough is modestly decreased at the UGA-C/U/A tetranucleotides at which tRNA^Trp^*[tW(CCA)G1]* serves as an rti-tRNA (Figure 4, compare columns 7 and 2) and largely unaffected at the UAA-N and UAG-C tetranucleotides at which tRNA^Tyr^*[tY(GUA)J2]* serves as a rti-tRNA (Figure 4, compare columns 9 and 4), when these tRNAs are overexpressed. The latter result suggests that U35 in tyrosine tRNA is not a substrate for pseudouridylation by PUS1, as was suggested earlier (41).

In contrast to this, we observe more robust rti-tRNA:tetranucleotide specific effects on readthrough in the *pus7Δ* background. In particular, readthrough caused by tRNA^Tyr^*[tY(GUA)J2]* at the UAA-N and UAG-C tetranucleotides is here diminished (Figure 4, compare columns 14, 9 and 4), consistent with the absence of the Ψ35 anticodon modification interfering with readthrough. tRNA^Trp^*[tW(CCA)G1]*, which does not normally contain pseudouridine modifications in the anticodon, is largely unaffected in its ability to promote readthrough at UGA-C/U/A tetranucleotides, when present at elevated levels (Figure 4, compare columns 12 and 2).

We next explored the effects of 2′-O-ribose methylation of tRNA^Trp^*[tW(CCA)G1]* by monitoring readthrough in a *trm7Δ* background. TRM7 is a 2′-O-ribose methyltransferase responsible for modifying tRNA^Phe^, tRNA^Leu^ and tRNA^Trp^ at positions C32 and C34, the latter being the third, wobble, position of the anticodon (41). Like the Ψ35 modification, methylation at C34 is thought to result in improved stacking with neighboring bases (44,45). Surprisingly, recent results suggest that UGA readthrough is unaffected in *trm7Δ* background, nor is the degree to which near-cognate tRNA accommodation results in incorporation of the corresponding amino acids tryptophan, cysteine and arginine (44). In fact, YARIS reveals that in a *trm7Δ* background, where methylation at C34 and C32 does not occur, readthrough by tRNA^Trp^*[tW(CCA)G1]* is increased at the UGA-C and UGA-A tetranucleotides (Figure 4, compare columns 17 and 2), whereas readthrough by other rti-tRNAs, which are not substrates of TRM7, remain unaffected.

At last, we also investigated the effects of deleting *MOD5*, which is responsible for the i^6^A modification at position 37 (3′ adjacent to the anticodon) in the rti-tRNAs tRNA^Tyr^ and tRNA^Cys^ (44,46). In the *mod5Δ* background, we observe a robust effect, suggesting that none of these tRNAs upon overexpression are able to induce readthrough at UGA stop for tRNA^Cys^*[tC(GCA)P1]* (Figure 4, compare columns 23 and 3) and UAA and UAG readthrough for tRNA^Tyr^*[tY(GUA)J2]* (Figure 4, compare columns 24 and 4). Nonetheless, we also observe a systematic decrease in readthrough levels under most conditions (Figure 4, compare columns 22–25 and 2–5), consistent with a general translational defect and complicating any inference of rti-tRNA:tetranucleotide specific defects. These effects, together with the other effects observed in the absence of specific tRNA modifications, are consistent with the idea that modifications within or adjacent to the anticodon most significantly affect the incorporation of the rti-tRNA at its corresponding stop tetranucleotide.

### YARIS reveals rti-tRNA: tetranucleotide-specific effects of aminoglycosides

**Figure 5.**
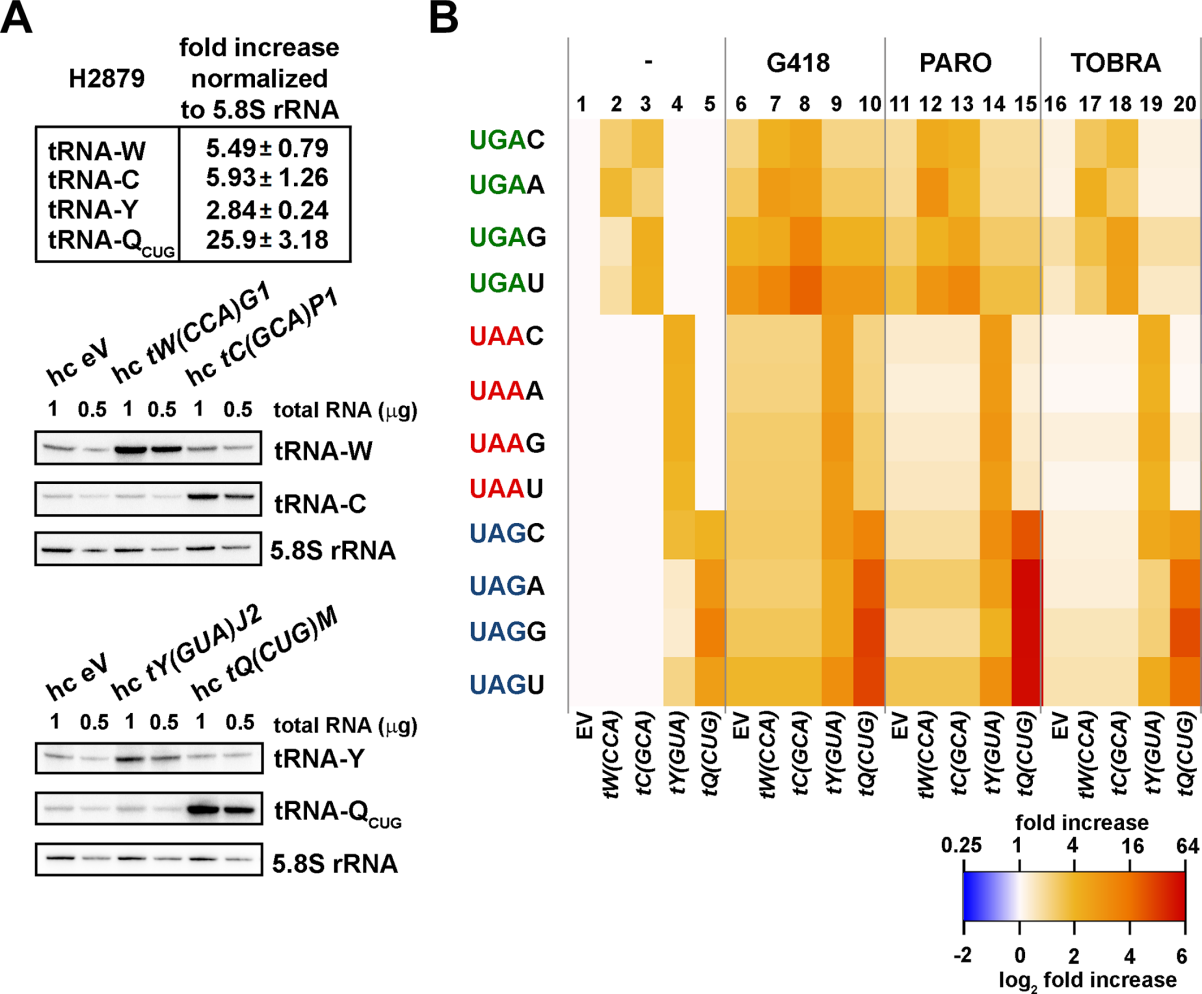
YARIS reveals rti-tRNA:tetranucleotide specific effects of aminoglycosides. (**A**) Quantification of cellular levels of four rti-tRNAs individually overexpressed in the H2879 strain. The upper panel summarizes fold-increases of overexpressed rti-tRNAs relative to an EV after normalization to the cellular level of 5.8S rRNA; the middle and bottom panels show the corresponding northern blots. Total RNAs were extracted from the H2879 strain bearing a high copy plasmid indicated at the top of each panel and 1 or 0.5 μg aliquots were loaded onto the Criterion Precast gels and subjected to northern blot with ^32^P-labeled probes shown on the right. Northern blots were quantified using the Quantity One program and the signals were first normalized to the control 5.8S rRNA. The resulting values obtained with cells bearing an EV (hc EV) were then set to 1.00 and those obtained with cells overexpressing individual rti-tRNAs were expressed relative to the hc EV control. Standard deviations from three individual experiments are given. (**B**) The Log2 fold increase heatmap of the readthrough induction in strains expressing various combinations of rti-tRNAs (columns) with stop codon tetranucleotides (rows) that were subjected to the indicated aminoglycoside treatments. In particular, the H2879 strain was transformed with either EV; hc tW(CCA)G1; hc tC(GCA)P1; hc tY(GUA)J2 or hc tQ(CUG)M, and the resulting transformants were grown in SD only (−), or supplemented for six hours with either G418 (**G418**; final concentration 50μg/ml); paromomycin (**PARO**; 200 μg/ml), or tobramycin (**TOBRA**; 400 μg/ml); and processed for stop codon readthrough measurements as described above. For each stop codon tetranucleotide (shown to the left) the readthrough values from untreated cells bearing the ‘EV’ were set to 1 (column 1).

We next employed YARIS to investigate the effects of three aminoglycosides previously shown to affect readthrough efficiencies: G418, paromomycin, and tobramycin (47,48). The Psi^−^ H2879 genetic background which displayed the highest basal readthrough efficiencies at UGAN tetranucleotides (30) was selected to perform these experiments. As with our previous experiments, we first confirmed the overexpression of the rti-tRNA by northern blot (Figure 5A, lower panels).

Before applying YARIS to interrogate readthrough in the presence of the aminoglycosides, we first confirmed that the pattern of readthrough effects we observe in the absence of drugs was similar to that observed in the PBH156 background. To create a more streamlined assay compatible with a high-throughput drug screening approach, we monitored readthrough levels in the presence of high levels of each rti-tRNA only at those stop tetranucleotides to which it is specific, and compared these to readthrough levels observed with the EV only (Figure 5B, compare column 1 with columns 2–5). Note that for combinations excluded from the analyses, we used the data obtained with the EV to visualize previously observed ‘no significant induction’ by tRNA overexpression. Despite the use of a distinct genetic background and other small differences in the experimental protocol applied here (6 h incubations, ± drug), the pattern of effects we observe is similar to that observed in the PBH156 background, highlighting the robustness of the rti-tRNA:tetranucleotide specificity rules and the YARIS protocol (e.g. compare Figure 5B, columns 1 and 4–5 with Figure 3B, columns 1 and 4–5).

In comparison to this pattern of effects, we observe a broad-based increase in the degree of readthrough in the presence of the aminoglycosides G418 and paromomycin. (Figure 5B, columns 6 and 11, respectively). And yet, the pattern of these effects is not uniform across the stop tetranucleotides. G418, which provokes the most severe increase in readthrough levels, increasing readthrough by at least 2-fold at all tetranucleotides, nonetheless provokes more dramatic effects at UAG-U, UGA-G and UGA-U, the latter being most sensitive to the drug, which increases readthrough 7.5-fold at this tetranucleotide. These effects were observed under milder treatment levels (50 μg/ml over 6 h) than those usually used in the literature. With the lone exception of the UAG-A tetranucleotide, paromomycin produces a similar pattern of effects (pronounced increase at UAG-U, UGA-G and UGA-U), but overall does not increase readthrough levels as dramatically. The fold increase at UAG-A is, however, as strong as with G418 (2.4-fold) (Figure 5B, column 11), pointing at a clear difference in tetranucleotide-specificity of these two RTIDs. Paromomycin treatment was performed as in our previous studies: 200 μg/ml over 6 h (30,31). In contrast to these effects, tobramycin only modestly affects readthrough levels between 1.3- and 1.7-fold at all tetranucleotides (Figure 5B, column 16). These modest effects result from the treatment condition (400 μg/ml over 6 h), which employed a concentration determined by previous studies to lie near the threshold concentration required for an observable effect (48). Nonetheless, these conditions result in a distinct induction pattern when compared to the other two aminoglycosides: the strongest of these modest effects by tobramycin is observed at the UGA-G tetranucleotide (Figure 5B, compare column 16 to 11 and 6).

We next asked whether these three drugs induce readthrough by enhancing the incorporation of rti-tRNAs at their corresponding stop codon tetranucleotides. In G418- and paromomycin-treated cells with elevated levels of tryptophan (Figure 5B, column 7 and 12) and cysteine tRNAs (Figure 5B, column 8 and 13), the pattern caused by the rti-tRNA-decoding rules and repeatedly observed in non-treated cells (Figure 5B, column 2 and 3) changes, suggesting that both aminoglycosides do not induce readthrough by promoting specific incorporation of rti-tRNAs. In fact, they modulate the preferential decoding by rti-tRNAs to some degree, most probably by destabilizing the ribosomal decoding site. For instance the most significant effect (an increase) was observed with G418 in combination with cysteine rti-tRNA at UGA-U (Figure 5B, in UGA-U row compare column 8 with 3 and 6). Similarly, tobramycin in these cells increases UGA-G readthrough, but only when tryptophan, and not cysteine, is overexpressed (Figure 5B, in UGA-G row compare column 17 with 2 and 16, and 18 with 3 and 16). Tryptophan does not function as an rti-tRNA at UGA-G (Figure 2) suggesting that tobramycin does not help rti-tRNAs to decode the stop codon either.

In cells overexpressing tRNA^Tyr^*[tY(GUA)J2]* (Figure 5B, columns 9, 14, and 19), none of the tested drugs had a significant impact on UAA-N tetranucleotides, as compared to non-treated cells (Figure 5B, column 4). However, we observe a level of readthrough induction at UAG-N similar to UAA-N (4- to 9- fold), suggesting that G418 and paromomycin treatments also modulate the tetranucleotide specificity for tyrosine (tobramycin displays a lot smaller effect, if any). In other words, the presence of aminoglycosides partially neutralize the decoding rules established for tRNA^Tyr^*[tY(GUA)J2]* as a near-cognate tRNA for UAG earlier; perhaps by promoting or impairing the action of rti-tRNAs at different stop codon tetranucleotides.

The largest observed impact of aminoglycoside induced readthrough was in the presence of elevated levels of tRNA^Gln^*[tQ(CUG)M]*, which results in severe increases in readthrough efficiency at the UAG-N tetranucleotides. This effect is most dramatic in the presence of paromomycin, raising readthrough levels (induction up to 57-fold) at the UAG-N tetranucleotides above those observed even in the presence of G418, which otherwise induces the largest general effects on readthrough (Figure 5B, compare columns 10 and 15). A similar effect can be observed in the presence of tobramycin as well. Although tobramycin alone produces only very modest effects on readthrough, in the presence of elevated levels of tRNA^Gln^*[tQ(CUG)M]* we observe a greater than 10-fold induction of readthrough at UAG-N tetranucleotides (Figure 5B, column 20). This effect is most pronounced at the UAG-G tetranucleotide, where we observe a 26-fold increase in readthrough levels approaching the level we observe in the presence of G418 (Figure 5B, column 10; 30-fold effect).

Taken together, these results point to both broad-based and rti-tRNA:tetranucleotide specific effects in the presence of readthrough-inducing aminoglycosides drugs. This complex portfolio of effects has implications for therapeutic approaches aimed at inducing readthrough at specific PTCs involved in human diseases.

## DISCUSSION

While faithful translation usually depends on the efficient recognition of stop codons by termination factors, ever-present competition for A-site binding by near-cognate tRNA can and does result in stop-codon readthrough. Recent work has elucidated several factors influencing the efficiency of stop-codon readthrough (13–17), such as the +4 nucleotide immediately following the stop codon (23–26) shown to determine the preference of each stop codon for specific near-cognate tRNA (28–31). The work of many labs thus began to delineate a set of specificity rules governing the efficiency of stop-codon readthrough at what is perhaps more accurately defined as a stop tetranucleotide.

In an effort to describe a more complete set of rules, we extended our prior work—which focused on the most readthrough-prone UGA-N tetranucleotide set—to interrogate the readthrough preferences of the remaining UAA-N and UAG-N tetranucleotide sets. As with our previous work, these experiments revealed the specificity of each stop tetranucleotide for a specific tRNA, and highlighted the distinct nature of these preferences. While the UAA-N tetranucleotides prefer the canonical wobble-mismatch near-cognate tRNA^Tyr^*[tY(GUA)J2]*, the UAG-N set favors tRNA^Gln^*[tQ(CUG)M]*, a non-canonical near-cognate presenting a mismatch at the first position (Figure 1). Even within tetranucleotide sets, preferences are highly specific: despite the preference of UAG-N for tRNA^Gln^*[tQ(CUG)M]*, UAG-C nonetheless incorporates tRNA^Tyr^*[tY(GUA)J2]* with relative efficiency, whereas the remaining UAG-N tetranucleotides do not. Taken together with our work and that of other labs, these results identify a subset of stop-codon near-cognate tRNAs that promote tetranucleotide-specific readthrough. We refer to these as readthrough-inducing tRNAs, or rti-tRNAs (Figure 2).

To more systematically interrogate the nature of this tetranucleotide specificity, we built upon our earlier work to develop a method to monitor readthrough efficiencies at the complete set of stop tetranucleotides in budding yeast, in the presence or absence of elevated levels of rti-tRNAs, and under a variety of environmental conditions (Figure 3). We call this method YARIS, and first demonstrated its utility by monitoring readthrough at all stop tetranucleotides in the presence of elevated levels of each of the rti-tRNA previously identified. This single-experiment recapitulates much of our previous work, and once again highlights the remarkable specificity observed at each tetranucleotide or with each rti-tRNA. This specificity is further underscored by our observation that no changes were observed when combinations of two rti-tRNAs, each with different stop codon specificity, were overexpressed. This is consistent with our interpretation that, even at elevated levels, rti-tRNA do not effectively compete at off-target tetranucleotides (Figure 3). The molecular determinants that enable this specific competition and more specifically enable distinct near-cognate tRNAs to function as rti-tRNAs whereas others do not, remain unclear.

We next employed YARIS to investigate whether or not discrete tRNA modifications contribute to this specificity. Our findings point to a role for modifications within or adjacent to the anticodon, in mediating efficient accommodation of near-cognate tRNA with mismatches at the first or third (wobble) position; however, not in the tetranucleotide-specific manner (Figure 4). In particular, deletion of *PUS7*, responsible for pseudouridylation of U35—position 2 of the anticodon—in tRNA^Tyr^*[tY(GUA)J2]* and other tRNAs, blocks the incorporation of tRNA^Tyr^*[tY(GUA)J2]* at UAA and UAG stop codons independently of the +4 nucleotide. This is consistent with the proposal that this modification improves stacking with neighboring base pairs (44,45). In the absence of the modification, efficient stacking with the matched pair at position one may be disrupted, exacerbating the penalty of the wobble mismatch in UAA:GUA and UAG:GUA pairs. Similarly, we observe the rti-tRNA:tetranucleotide-specific loss of readthrough efficiency upon deleting *MOD5*, which normally targets the anticodon adjacent position 37 of tRNA^Cys^*[tC(GCA)P1* and tRNA^Tyr^*[tY(GUA)J2]*. In contrast, deletion of *TRM7*, which modifies C32 and C34—the third (wobble) position of the anticodon—of tRNA^Trp^*[tW(CCA)G1]* by 2′O-methylation, actually increases readthrough efficiency at the UGA-C and UGA-A tetranucleotide targets of this rti-tRNA. This result is in contrast to previous results (45), as well as the suggestion that 2′-O-methylation at C34 improves base stacking by rigidifying the duplex (44,45). Another possibility is that steric constraints imposed by the modification magnify the thermodynamic penalty of a wobble mismatch, and thus the absence of the modification enables the adoption a stacking geometry more forgiving a C_34_:A pair at this position. Finally, whereas the deletion of *PUS7, TRM7* and *MOD5*—which specifically target positions within or adjacent to the anticodon—produces specific effects, deletion of *PUS1*, which serves as a general pseudouridine synthase targeting sites throughout tRNA, does not. This observation is consistent with the current model in which local interactions between the stop tetranucleotide and the anticodon stem of the tRNA, and not global structural features of the tRNA themselves, are believed to be responsible for mediating accommodation of rti-tRNA. Nonetheless, more careful investigations of the role played by general tRNA dynamics and structure is required to determine this unequivocally.

Because of the role that premature termination codons play in human disease (9,10) and the emerging interest in drug-induced readthrough as a therapeutic approach (49), we next employed YARIS to investigate the interplay between selected aminoglycoside RTIDs and elevated levels of rti-tRNA. These experiments revealed that, while G418, paromomycin and tobramycin affect general readthrough to varying degrees, they also induce tetranucleotide-specific effects that are further modulated under increased levels of the rti-tRNA specific to those tetranucleotides. All three drugs induce pronounced effects at UAG-U, UGA-G and UGA-U tetranucleotides, with those observed in the presence of G418 being the most severe. Nonetheless, in the presence of elevated levels of tRNA^Gln^*[tQ(CUG)M]*, readthrough at the UAG-N tetranucleotide set is dramatically increased in the presence of all of these drugs. In fact, despite producing more modest effects in the absence of elevate rti-tRNA levels, paromomycin is strongly potentiated under these conditions, resulting in the highest readthrough levels observed in this experiment. Tobramycin, which on its own induces only modest readthrough effects, is similarly activated at high tRNA^Gln^*[tQ(CUG)M]* levels. Taken together, the present and previous works highlight the important role that rti-tRNA levels play in both spurious and programmed readthrough events. Previous observations of tissue and organism-specific variations in both readthrough (1) and tRNA isoform levels (50) suggest a possible regulatory role for cellular levels of this functional group of tRNAs (rti-tRNAs) in fine-tuning the relative levels of C-terminally distinct protein isoforms and thereby expanding proteomic versatility.

In the context of the tetranucleotide-specific effects we observed for RTIDs and the specificity of rti-tRNA:tetranucleotide recognition we and others have described, these observations suggest that personalized approaches to the treatment of PTC-induced diseases may be advantageous. Matching PTC tetranucleotides with corresponding RTIDs at non-toxic concentrations and a high dosage of corresponding exogenous rti-tRNAs administered in tandem might enable the maximization of targeted readthrough while minimizing off-target miscoding. In this regard, as exemplified here, the YARIS system developed by us could be leveraged to test whether existing readthrough drugs interfere with the general rules of stop codon decoding by the rti-tRNA in the comprehensive manner to assess their effectiveness and selectivity. In addition, YARIS could also expedite future studies aimed at identifying novel and tetranucleotide-specific readthrough drugs.

Programmed stop codon readthrough is a major regulatory mechanism of the termination phase of the protein synthesis that plays a significant role in the physiology of the cell, as evidenced by a broad spectrum of genes with diverse functions possessing stop codons in a predictably high-readthrough nucleotide context in numerous organisms (51). As an example, our computational search identified 30 such genes from the entire *S. cerevisiae* genome (Supplementary Figure S10 and Excel File). Hence, thanks to its complexity, we encourage our colleagues using YARIS as a generally used assay for determining the readthrough potential of all candidate genes across species. In case of yeast, it could be for instance used for testing strains with mutations of proteins or RNA involved not only in the termination phase, but also in general translation to exclude that the otherwise observed phenotype is not merely a downstream effect of the misbalanced translation termination.

Taken together, our results provide key mechanistic insights into the specific recognition of stop tetranucleotides by rti-tRNAs and how this recognition is affected by tRNA modifications and RTIDs. More broadly, our study demonstrates the utility of YARIS to systematically interrogate readthrough at multiple stop contexts and under a broad array of genetic and environmental conditions.

## Supplementary Material

gkz346_Supplemental_Files
