# Supplementary material for: Yeast applied readthrough inducing system (YARIS): an invivo assay for the comprehensive study of translational readthrough
Source: Nucleic Acids Res. 2019 May 9;47(12):6339–50. doi: 10.1093/nar/gkz346 (PMC6614816; doi:10.1093/nar/gkz346)
Supplement: gkz346_Supplemental_Files [file gkz346_supplemental_files.zip › Beznoskova et al 2018_SupMat_R2.pdf]

SUPPLEMENTARY MATERIAL

SUPPLEMENTARY MATERIALS AND METHODS

Construction of yeast strains and plasmids

List of all strains used throughout this study can be found in Table S1.

To create PBH156 strain; PBH140 was transformed with YCp22-g/TIF35-screen and the Uracil auxotrophy was regained by growing the cells on SD plates containing 5-fluoro-orotic acid (5-FOA) resulting in loss of YEp-TIF35-U plasmid.

List of all plasmids and PCR primers used throughout this study can be found in Tables S2 and S3, respectively.

PBB157, PBB158; PBB159; PBB160; PBB161; and PBB162 were constructed by inserting the 4567-bp *Alw*NI-*Nsi*I fragment from pDB689; pDB718; pDB730; pDB731; pDB725 and pDB727; respectively, into YEplac181 digested by *Alw*NI-*Nsi*I.

PBB146 was created by inserting the *Bam*HI-*Xho*I digested PCR product obtained with primers PB130 and PB131 using genomic DNA obtained from yeast strain H464 as template into *Bam*HI-*Xho*I digested pTH335.

PBB147 was created by inserting the *Bam*HI-*Xho*I digested PCR product obtained with primers PB132 and PB133 using genomic DNA obtained from yeast strain H464 as template into *Bam*HI-*Xho*I digested pTH335.

PBB148 was created by inserting the *Bam*HI-*Xho*I digested PCR product obtained with primers PB134 and PB135 using genomic DNA obtained from yeast strain H464 as template into *Bam*HI-*Xho*I digested pTH335.

PBB149 was created by inserting the *Bam*HI-*Xho*I digested PCR product obtained with primers PB136 and PB137 using genomic DNA obtained from yeast strain H464 as template into *Bam*HI-*Xho*I digested pTH335.

PBB150 was created by inserting the *Bam*HI-*Xho*I digested PCR product obtained with primers PB140 and PB141 using genomic DNA obtained from yeast strain H464 as template into *Bam*HI-*Xho*I digested pTH335.

PBB156 was created by inserting the *Bam*HI-*Xho*I digested PCR product obtained with primers PB142 and PB143 using genomic DNA obtained from yeast strain H464 as template into *Bam*HI-*Xho*I digested pTH335.

PBB134 was created by inserting the *Sal*I-*Xho*I digested fragment from pSZ61 into *Sal*I-*Xho*I digested pTH335.

ZPB30 was created by inserting the *Bam*HI-*Sac*I digested PCR product obtained with primers PB223 and PB95 using PBB97 as template into *Bam*HI-*Sac*I digested PBB90.

ZPB31 was created by inserting the *Bam*HI-*Sac*I digested PCR product obtained with primers PB224 and PB141 using PBB150 as template into *Bam*HI-*Sac*I digested PBB90.

ZPB32 was created by inserting the *Bam*HI-*Sac*I digested PCR product obtained with primers PB222 and PB106 using pTH335 as template into *Bam*HI-*Sac*I digested PBB90.

ZPB33 was created by inserting the *Bam*HI-*Sac*I digested PCR product obtained with primers PB222 and PB106 using pTH335 as template into *Bam*HI-*Sac*I digested PBB97.

ZPB34 was created by inserting the *Bam*HI-*Sac*I digested PCR product obtained with primers PB223 and PB95 using PBB97 as template into *Bam*HI-*Sac*I digested PBB150.

ZPB35 was created by inserting the *Bam*HI-*Sac*I digested PCR product obtained with primers PB222 and PB106 using pTH335 as template into *Bam*HI-*Sac*I digested PBB150.

PBB81 was created by inserting the *Sal*I-*Not*I digested PCR product obtained with primers TIF32 and PBRFNotI using pTH477 as template into *Sal*I-*Not*I digested pTH477.

SUPPLEMENTARY REFERENCES

1. Beznoskova, P., Wagner, S., Jansen, M.E., von der Haar, T. and Valasek, L.S. (2015) Translation initiation factor eIF3 promotes programmed stop codon readthrough. *Nucleic Acids Res*, **43**, 5099-5111.
2. Beznoskova, P., Gunisova, S. and Valasek, L.S. (2016) Rules of UGA-N decoding by near-cognate tRNAs and analysis of readthrough on short uORFs in yeast. *RNA*, **22**, 456-466.
3. Lowe, T.M. and Chan, P.P. (2016) tRNAscan-SE On-line: integrating search and context for analysis of transfer RNA genes. *Nucleic Acids Res*, **44**, W54-57.
4. Pineyro, D., Torres, A.G. and de Pouplana, L.R. (2014) *Biogenesis and Evolution of Functional tRNAs*. Springer, Cham.
5. Nielsen, K.H., Szamecz, B., Valasek, L.J., A., Shin, B.S. and Hinnebusch, A.G. (2004) Functions of eIF3 downstream of 48S assembly impact AUG recognition and GCN4 translational control. *EMBO J.*, **23**, 1166-1177.
6. Cuchalová, L., Kouba, T., Herrmannová, A., Danyi, I., Chiu, W.-I. and Valášek, L. (2010) The RNA Recognition Motif of Eukaryotic Translation Initiation Factor 3g (eIF3g) Is Required for Resumption of Scanning of Posttermination Ribosomes for Reinitiation on GCN4 and Together with eIF3i Stimulates Linear Scanning. *Mol Cell Biol*, **30**, 4671-4686.
7. Keeling, K.M., Lanier, J., Du, M., Salas-Marco, J., Gao, L., Kaenjak-Angeletti, A. and Bedwell, D.M. (2004) Leaky termination at premature stop codons antagonizes nonsense-mediated mRNA decay in S. cerevisiae. *RNA*, **10**, 691-703.
8. Gietz, R.D. and Sugino, A. (1988) New yeast-Escherichia coli shuttle vectors constructed with in vitro mutagenized yeast genes lacking six-base pair restriction sites. *Gene*, **74**, 527-534.
9. Leidel, S., Pedrioli, P.G., Bucher, T., Brost, R., Costanzo, M., Schmidt, A., Aebersold, R., Boone, C., Hofmann, K. and Peter, M. (2009) Ubiquitin-related modifier Urm1 acts as a sulphur carrier in thiolation of eukaryotic transfer RNA. *Nature*, **458**, 228-232.

SUPPLEMENTARY FIGURE LEGENDS

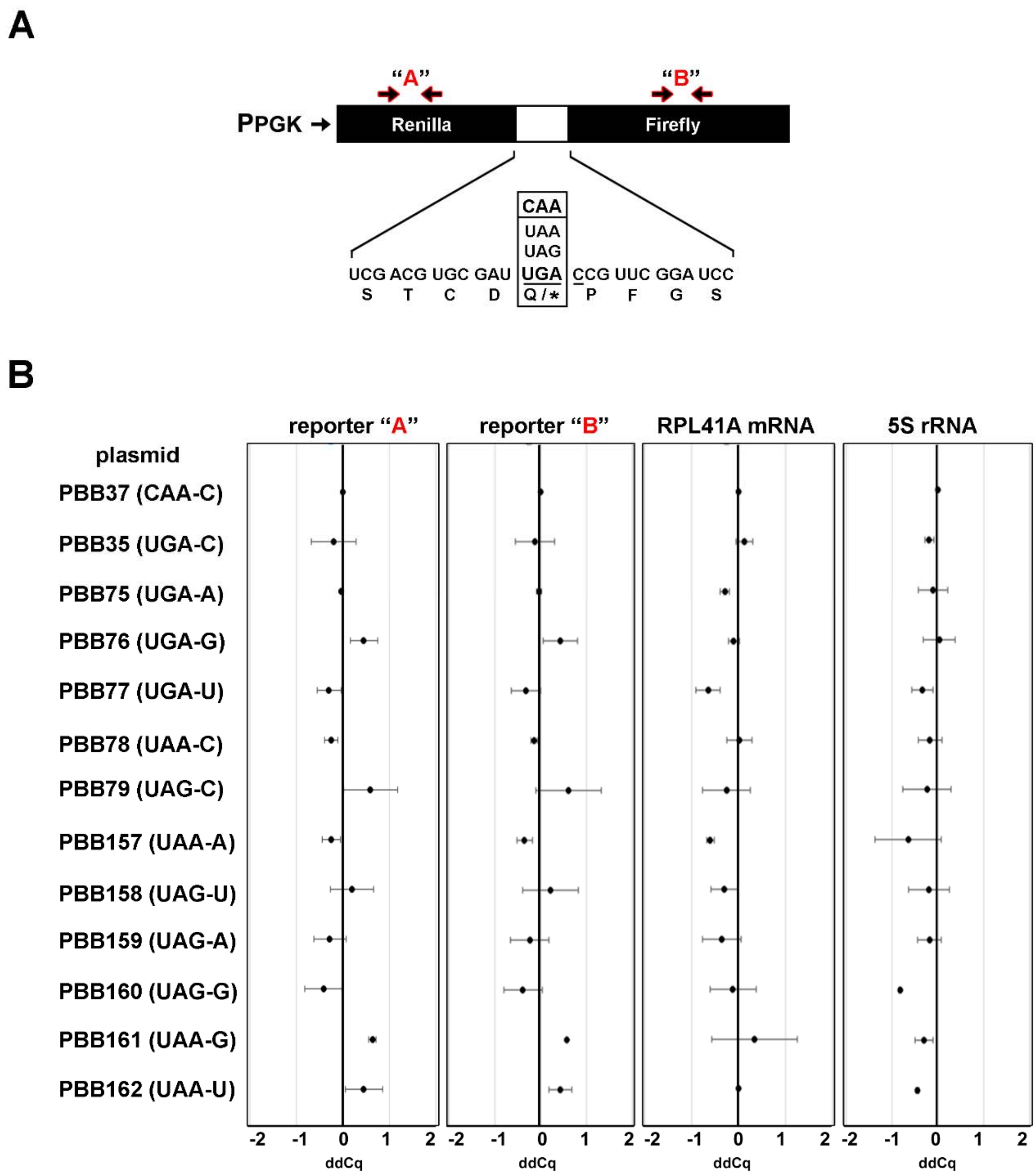

Figure S1. The mRNA levels of the given reporters bearing the stop signal in-between renilla and firefly genes do not differ from the CAA sense codon control. (**A**) Schematics of the reporters with two sets of qPCR primers "A" and "B". (**B**) The mRNA levels in the PBH156 strain expressing given reporters were assessed by quantitative PCR. Plots represent the result of three independent experiments ± SD. The ddCq value displays the threshold cycle normalized to the reference 5S rRNA and to CAA-C control strain. The ddCq values are in log2 scale. A ddCq of 0 indicates no change to to CAA sense control; ddCq = 1 or = -1 indicates a drop of 50% or doubling compared to CAA sense control, respectively.

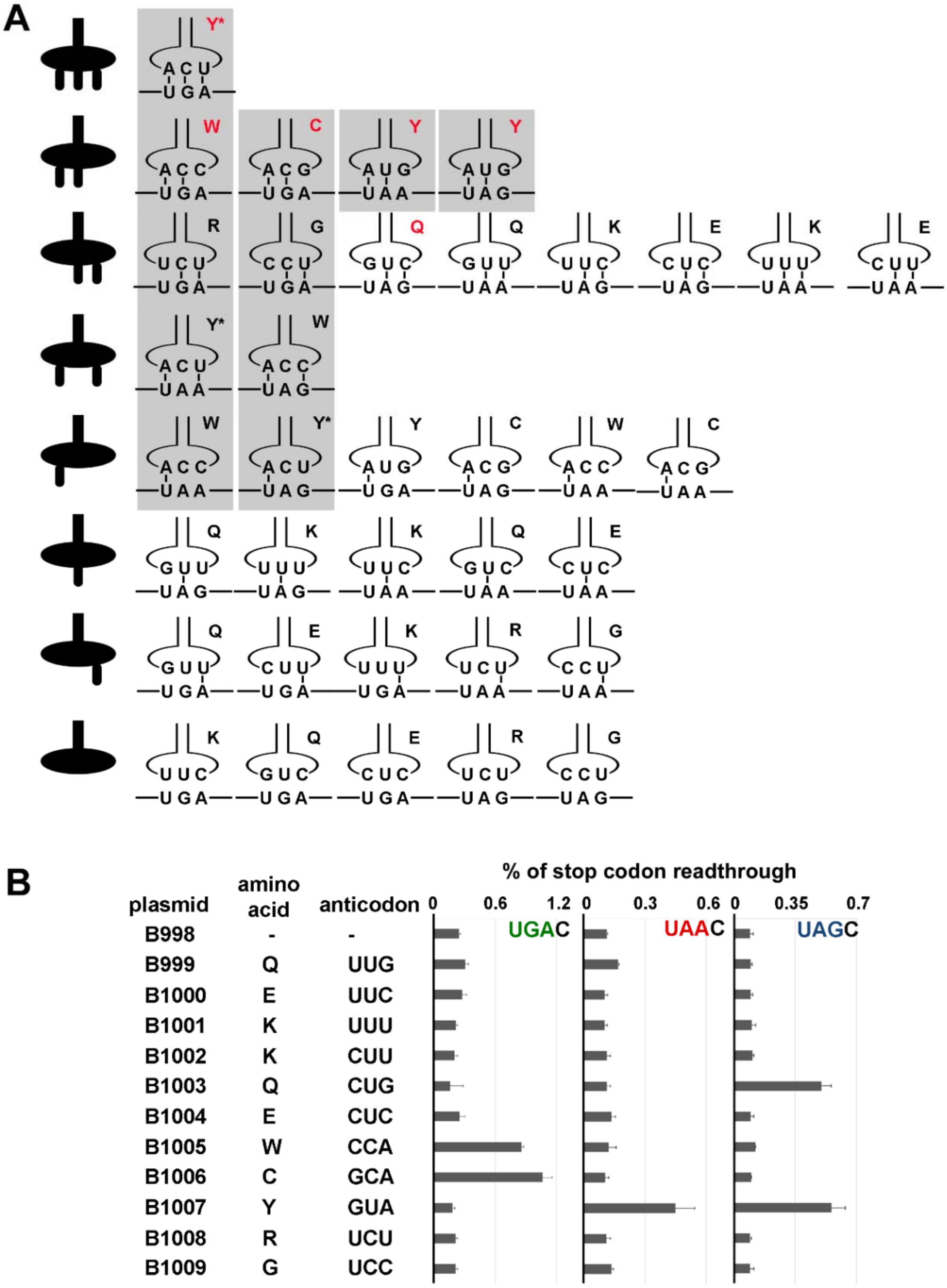

Figure S2. Induction of stop codon readthrough by overexpression of various tRNAs (**A**) Schematics of anticodon-stop combinations tested previously (in grey) (1,2) and in this study (in white). tRNA specifically inducing readthrough are labeled in red. Asterisk indicates a suppressor tyrosine tRNA tY(ACU) that is a result of a spontaneous mutation and thus does not naturally exist in the yeast genome (**B**) PBH156 was transformed with the given plasmids and the resulting transformants were grown and processed for stop codon readthrough measurements as described in Figure **1D.**

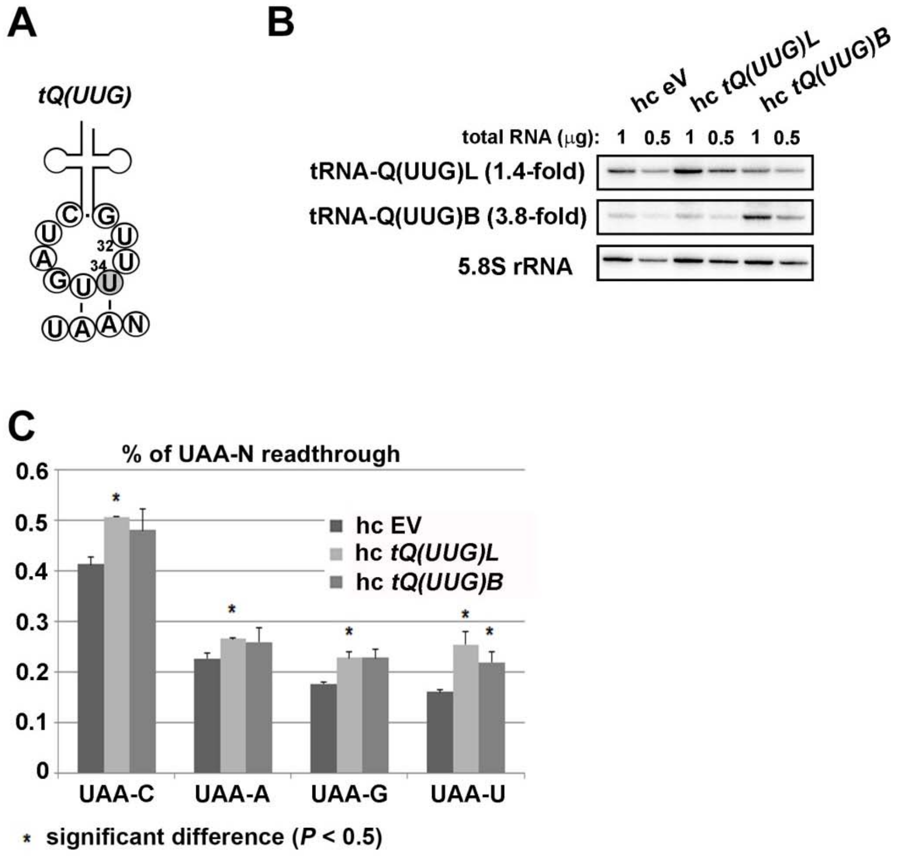

Figure S3. The glutamine tRNA tQ(UUG) does not serve as rti-tRNA at the UAA stop codon. (**A**) A schematic of the Gln tRNA base-paring with the UAA-N stop codon tetranucleotide. Only the nucleotides of the anticodon loop are shown with the third stop codon base N34 (in grey) and N32 indicated. (**B**) Increased gene dosage of tQ(UUG)L or tQ(UUG)B tRNAs increases their cellular levels *in vivo*. Total RNAs were extracted from the PBH156 strain bearing a plasmid indicated at the top of each panel and 1 µg or 0.5 µg aliquots were loaded onto the Criterion Precast gels and subjected to Northern blotting with ^{32}P-labelled probes shown on the left. (**C**) Increased gene dosage of the indicated Gln tRNAs shows little to none effect on readthrough at the UAA-N tetranucleotides. The PBH156 was transformed with either empty vector (EV), high copy (hc) tQ(UUG)L, or hc tQ(UUG)B and the resulting transformants were grown and processed for the stop codon readthrough measurements as described in Figure **1C.**

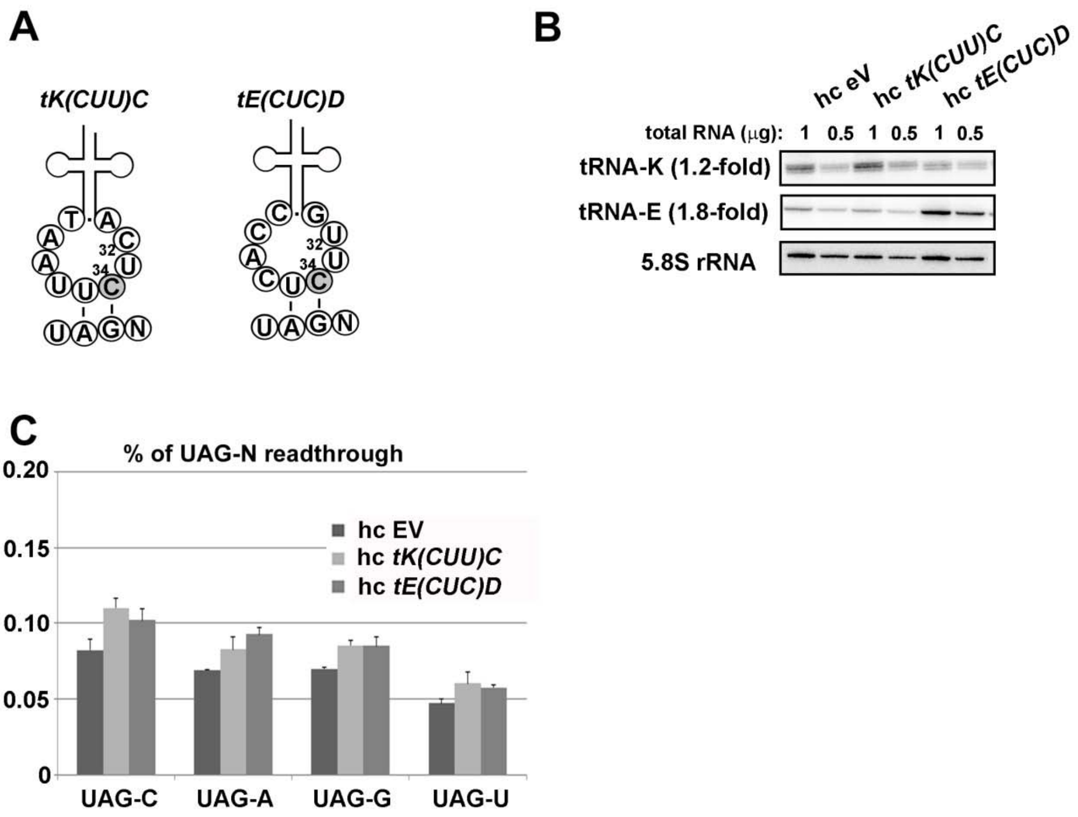

Figure S4. Increased gene dosage of lysine or glutamate tRNAs does not affect readthrough at any UAG-N tetranucleotide. (**A**) Schematics of Lys and Glu tRNAs base-paring with the UAGN stop codon tetranucleotide. Only the nucleotides of the anticodon loop are shown with the third stop codon base N34 (in grey) and N32 indicated. (**B**) Increased gene dosage of tK(CUU)D or tE(CUC)D tRNAs increases their cellular levels *in vivo*. Total RNAs were extracted from the PBH156 strain bearing a plasmid indicated at the top of each panel and 1 μg or 0.5 μg aliquots were loaded onto the Criterion Precast gels and subjected to Northern blotting with ^{32}P-labelled probes shown on the left. (**C**) Increased gene dosage of Lys or Glu tRNAs does not affect readthrough at any UAG-N tetranucleotide. The PBH156 strain was transformed with either empty vector (EV), high copy (hc) tK(CUU)C, or hc tE(CUC)D and the resulting transformants were grown and processed for stop codon readthrough measurements as described in Figure **1D**.

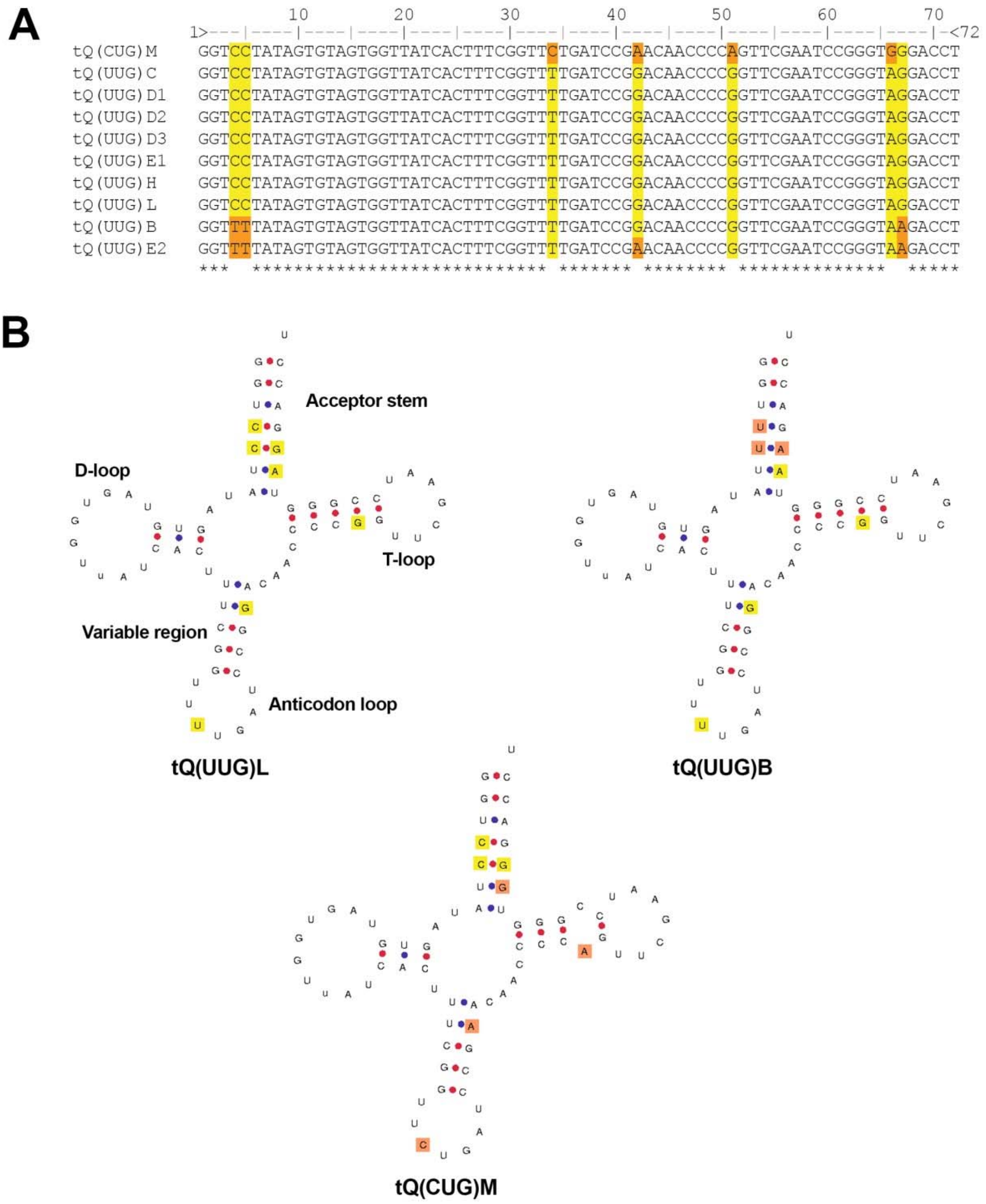

Figure S5. Variability among the *S. cerevisiae* glutamine tRNA primary sequences. (**A**) A multiple sequence alignment of *S. cerevisiae* glutamine tRNA isotypes. Virtually all sequences are identical except for variants M, B, and E2, respectively. Positions of the consensus sequence divergence are highlighted in yellow, base variations in orange. Sequences were obtained from GtRNAdb (3). (**B**) Schematics illustrating differences in the primary and secondary structures among all three glutamine tRNAs of interest. A variant L represents the consensus sequence, a variant B contains mutations in the Acceptor stem region, and a variant M contains the CUG anticodon sequence; color-coding as in part A. The tRNA secondary structure predictions were adapted from GtRNAdb (3).

A

```
                     10        20        30        40        50        60        70        80        90        100
            1>---|----|----|----|----|----|----|----|----|----|----|----|----|----|----|----|----|----|----|----|----|<106
tW(CCA)G1   GAAGCGGTGGCTCAATGGTAGAGCTTTCGACTCCAAttaaatcttggaaattccacggaataagattgcaATCGAAGGGtTGCAGGTTCAATTCCTGTCCGTTTCA
tW(CCA)G2   GAAGCGGTGGCTCAATGGTAGAGCTTTCGACTCCAAttaaatcttggaaattccacggaataagattgcaATCGAAGGGtTGCAGGTTCAATTCCTGTCCGTTTCA
tW(CCA)J    GAAGCGGTGGCTCAATGGTAGAGCTTTCGACTCCAAttaaatcttggaaattccacggaataagattgcaATCGAAGGGtTGCAGGTTCAATTCCTGTCCGTTTCA
tW(CCA)K    GAAGCGGTGGCTCAATGGTAGAGCTTTCGACTCCAAttaaatcttggaaattccacggaataagattgcaATCGAAGGGtTGCAGGTTCAATTCCTGTCCGTTTCA
tW(CCA)M    GAAGCGGTGGCTCAATGGTAGAGCTTTCGACTCCAAttaaatcttggaaattccacggaataagattgcaATCGAAGGGtTGCAGGTTCAATTCCTGTCCGTTTCA
tW(CCA)P    GAAGCGGTGGCTCAATGGTAGAGCTTTCGACTCCAAttaaatcttggaaattccacggaataagattgcaATCGAAGGGtTGCAGGTTCAATTCCTGTCCGTTTCA
            **********************************************************************************************************
                                                        intron
```

B

```
                     10        20        30        40        50        60        70
            1>---|----|----|----|----|----|----|----|----|----|----|----|----|----|-<72
tC(GCA)P1   GCTCGTATGGCGCAGTGGTAGCGCAGCAGATTGCAAATCTGTTGGtCCTTAGTTCGATCCTGAGTGCGAGCT
tC(GCA)P2   GCTCGTATGGCGCAGTGGTAGCGCAGCAGATTGCAAATCTGTTGGtCCTTAGTTCGATCCTGAGTGCGAGCT
tC(GCA)B    GCTCGTATGGCGCAGTGGTAGCGCAGCAGATTGCAAATCTGTTGGtCCTTAGTTCGATCCTGAGTGCGAGCT
tC(GCA)G    GCTCGTATGGCGCAGTGGTAGCGCAGCAGATTGCAAATCTGTTGGtCCTTAGTTCGATCCTGAGTGCGAGCT
            ************************************************************************
```

C

```
                     10        20        30        40        50        60        70        80
            1>---|----|----|----|----|----|----|----|----|----|----|----|----|----|----|----|----|---<89
tY(GUA)J2   CTCTCGGTAGCCAAGTTGGTttAAGGCGCAAGACTGTAAtttatcactacgaaATCTTGAGAtCGGGCGTTCGACTCGCCCCCGGGAGA
tY(GUA)D    CTCTCGGTAGCCAAGTTGGTttAAGGCGCAAGACTGTAAtttatcactacgaaATCTTGAGAtCGGGCGTTCGACTCGCCCCCGGGAGA
tY(GUA)O    CTCTCGGTAGCCAAGTTGGTttAAGGCGCAAGACTGTAAtttatcactacgaaATCTTGAGAtCGGGCGTTCGACTCGCCCCCGGGAGA
tY(GUA)J1   CTCTCGGTAGCCAAGTTGGTttAAGGCGCAAGACTGTAAtttaccactacgaaATCTTGAGAtCGGGCGTTCGACTCGCCCCCGGGAGA
tY(GUA)F1   CTCTCGGTAGCCAAGTTGGTttAAGGCGCAAGACTGTAAtttaccactacgaaATCTTGAGAtCGGGCGTTCGACTCGCCCCCGGGAGA
tY(GUA)F2   CTCTCGGTAGCCAAGTTGGTttAAGGCGCAAGACTGTAAtttaccactacgaaATCTTGAGAtCGGGCGTTCGACTCGCCCCCGGGAGA
tY(GUA)M1   CTCTCGGTAGCCAAGTTGGTttAAGGCGCAAGACTGTAAtttaccactacgaaATCTTGAGAtCGGGCGTTCGACTCGCCCCCGGGAGA
tY(GUA)M2   CTCTCGGTAGCCAAGTTGGTttAAGGCGCAAGACTGTAAtttaccactacgaaATCTTGAGAtCGGGCGTTCGACTCGCCCCCGGGAGA
            ******************************************** ********************************************
                                                 intron
```

Figure S6: Variability among the *S. cerevisiae* primary sequences of tryptophan (**A**); **cysteine** (**B**) **and tyrosine tRNAs** (**C**). Multiple sequence alignments of the tested rti-RNAs (always on top) with the remaining isotypes occurring in the *S. cerevisiae* genome. Please note that virtually all sequences are identical within the particular group of the isoacceptor tRNA. Sequences were obtained from GtRNAdb (3).

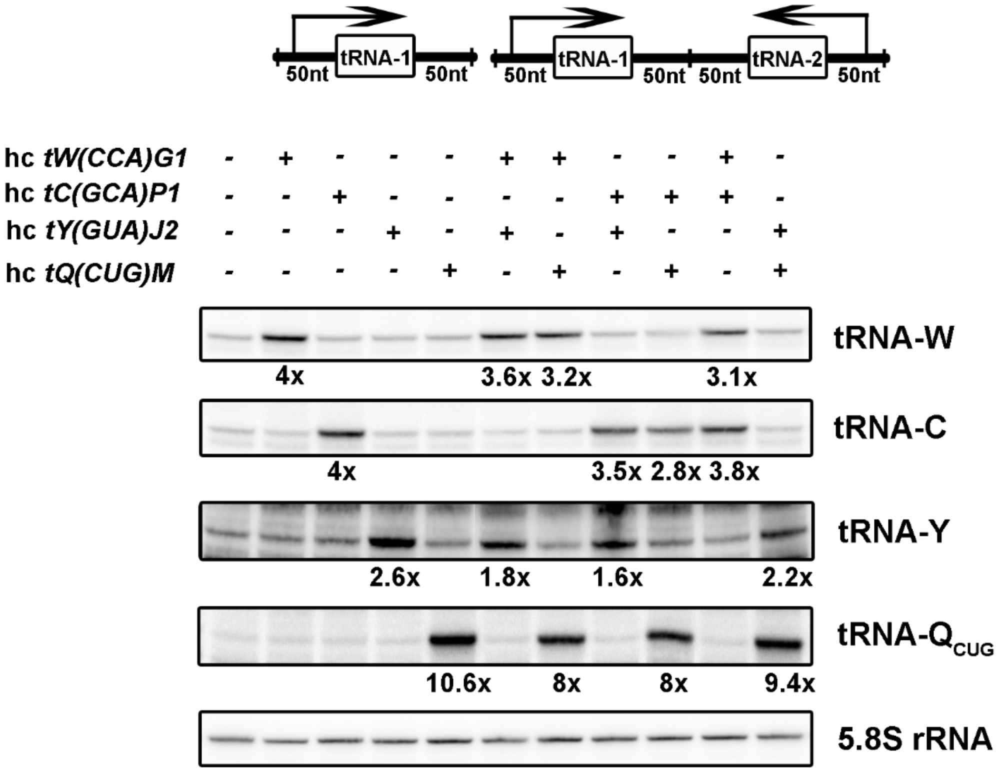

Figure S7: Increased gene dosage of single tRNAs increases their cellular level to a higher extend than when in combination with other tRNAs in high copy. Total RNA was extracted from the PBH156 strain bearing a plasmid indicated at the top of each panel, 2µg aliquots were loaded onto the Criterion Precast gels and subjected to Northern blotting with ^{32}P-labelled probes shown on the right. Fold increases relative to the empty vector control were calculated from two independent experiments.

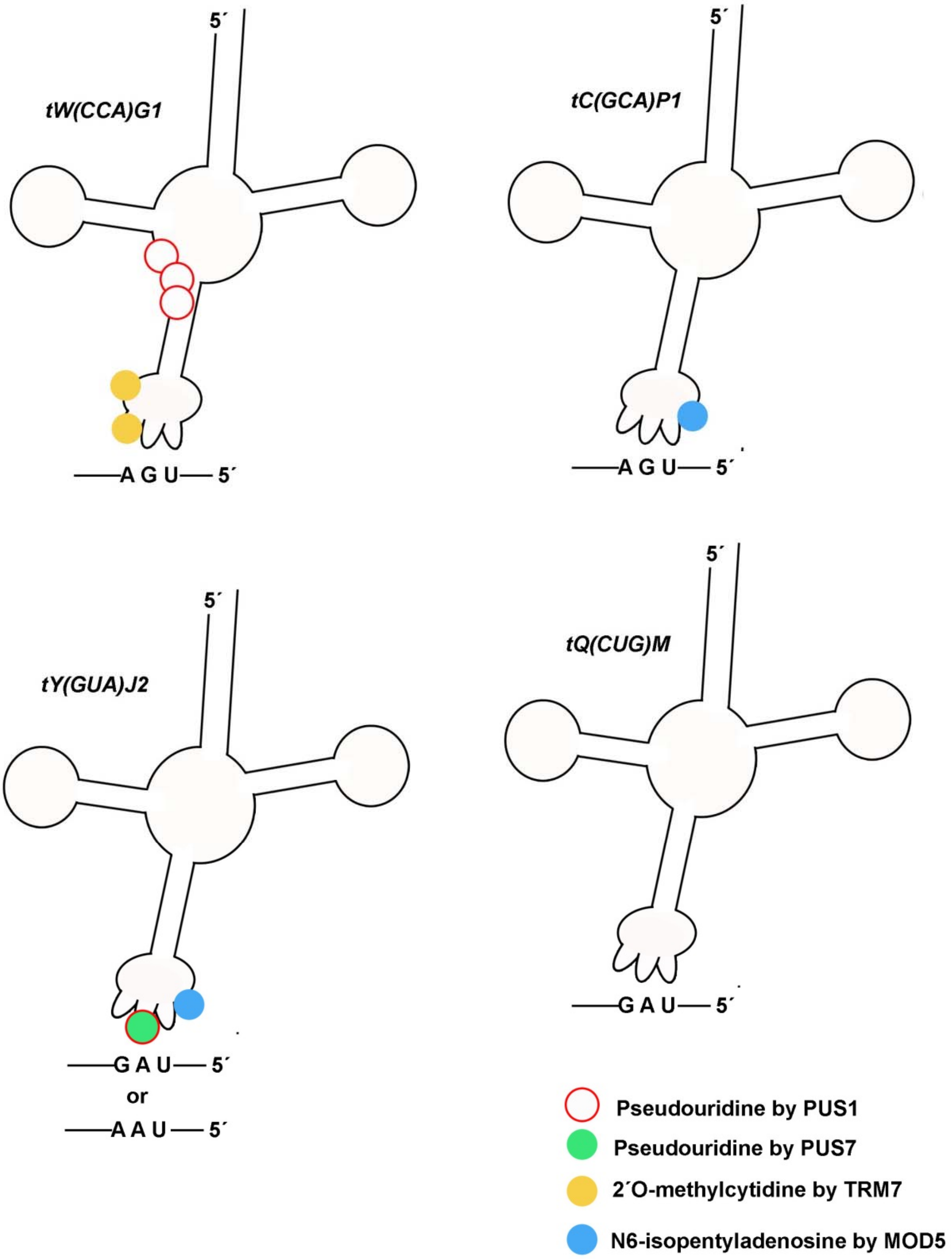

Figure S8. Selected modifications of all rti-tRNAs. Schematics of the rti-tRNAs identified here with the indicated positions that are the subject of specified modifications by *PUS1, PUS7, TRM7* and *MOD5* (adapted from (4)).

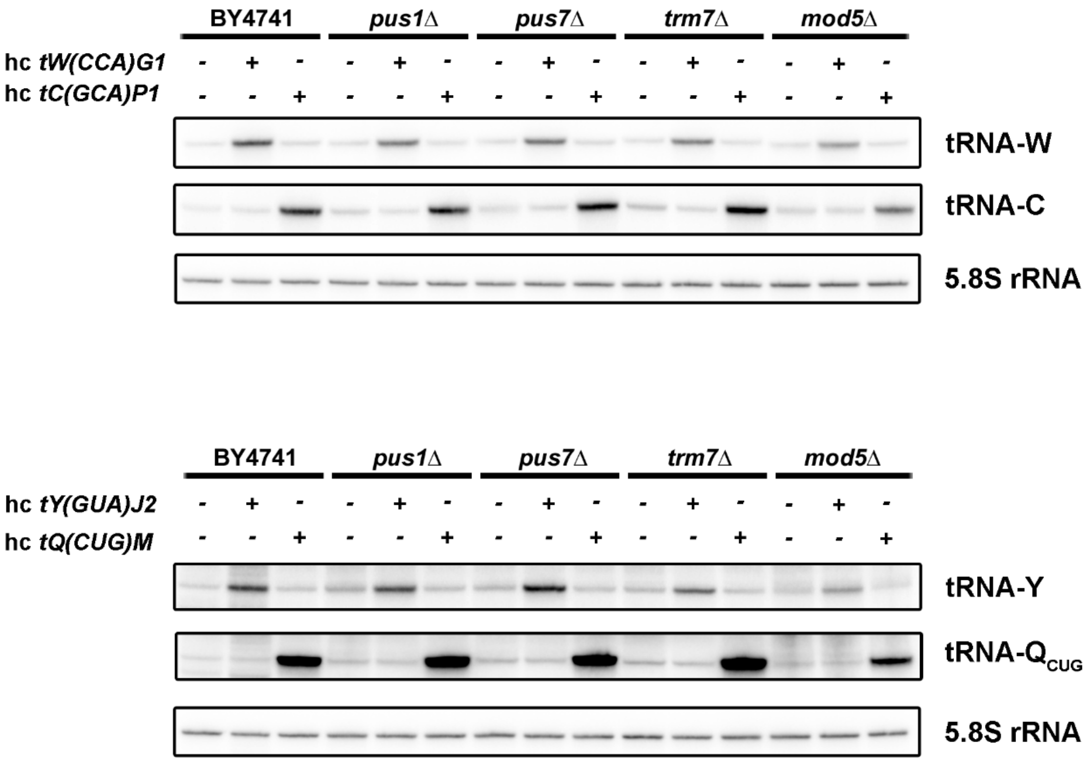

Figure S9: Increased gene dosage of selected tRNAs increases their cellular levels in strains individually deleted for genes encoding indicated tRNA modifying enzymes. Total RNA was extracted from the strains bearing a plasmid indicated at the top of each panel, 2μg aliquots were loaded onto the Criterion Precast gels and subjected to Northern blotting with ^{32}P-labelled probes shown on the right.

	gene name	systematic name	biological process	DNA stop-context	original stop	6nt context	readthrough (fold change)*	reference
1	YBR079C RPG1 SGDID:S00000028	YBR079C	translation	TAACAGACA	UAA	CAGACA	**8.1**	this study
2	YBR223C TDP1 SGDID:S00000042	YBR223C	DNA repair	TAACAAGTA	UAA	CAAGTA	nd	
3	YDL105W NSE4 SGDID:S00000226	YDL105W	DNA repair	TAACAGCGA	UAA	CAGCGA	nd	
4	YDR190C RVB1 SGDID:S00000259	YDR190C	transcription	TAACAGTGA	UAA	CAGTGA	nd	
5	YDR251W PAM1 SGDID:S00000265	YDR251W	pseudohyphal growth	TAACAAATA	UAA	CAAATA	nd	
6	YER038C KRE29 SGDID:S00000084	YER038C	DNA repair	TAACAGTCA	UAA	CAGTCA	nd	
7	YIL065C FIS1 SGDID:S00000132	YIL065C	fission of organels	TAACAAGCA	UAA	CAAGCA	nd	
8	YJL176C SWI3 SGDID:S00000371	YJL176C	transcription	TAACAATGA	UAA	CAATGA	nd	
9	YKL032C IXR1 SGDID:S00000151	YKL032C	cellular response of hypoxia	TAACAAATA	UAA	CAAATA	nd	
10	YKL038W RGT1 SGDID:S00000152	YKL038W	glucose metabolic process	TGACAGGTA	UGA	CAGGTA	nd	
11	YMR191W SPG5 SGDID:S00000480	YMR191W	proteasome assembly	TAGCAAAGA	UAG	CAAAGA	nd	
12	YNL047C SLM2 SGDID:S00000499	YNL047C	cytoskeleton organisation	TGACAAGTA	UGA	CAAGTA	nd	
13	YNL049C SFB2 SGDID:S00000499	YNL049C	intracelular trafic	TAACAATCA	UAA	CAATCA	nd	
14	YNL214W PEX17 SGDID:S00000515	YNL214W	targeting to peroxisomes	TAACAATCA	UAA	CAATCA	nd	
15	YNL269W BSC4 SGDID:S00000521	YNL269W	unknown	TGACAACTA	UGA	CAACTA	**22.6**	Beznoskova et al. 2015
16	YPR040W TIP41 SGDID:S00000624	YPR040W	signal transduction	TAACAATCA	UAA	CAATCA	nd	
17	YBR069C TAT1 SGDID:S00000027	YBR069C	amino acid transport	TAACAGCAA	UAA	CAGCAA	nd	
18	YDL088C ASM4 SGDID:S00000224	YDL088C	nucleopore organisation	TAACAGAAA	UAA	CAGAAA	nd	
19	YDR219C MFB1 SGDID:S00000262	YDR219C	mitochondrion organisation	TAACAGAAA	UAA	CAGAAA	nd	
20	YDR448W ADA2 SGDID:S00000285	YDR448W	chromatin organisation	TAACAAAAA	UAA	CAAAAA	nd	
21	YEL025C YEL025C SGDID:S00000075	YEL025C	unknown	TGACAATAA	UGA	CAATAA	nd	
22	YJL057C IKS1 SGDID:S00000359	YJL057C	protein phosphorylation	TAACAATAA	UAA	CAATAA	nd	
23	YJL102W MEF2 SGDID:S00000363	YJL102W	mitochondrial translation	TAGCAAAAA	UAG	CAAAAA	nd	
24	YLR074C BUD20 SGDID:S00000406	YLR074C	bud site selection	TAACAGTAA	UAA	CAGTAA	nd	
25	YLR245C CDD1 SGDID:S00000423	YLR245C	cytidine catabolic process	TAGCAGCAA	UAG	CAGCAA	nd	
26	YLR274W MCM5 SGDID:S00000426	YLR274W	DNA repair	TGACAACAA	UGA	CAACAA	nd	
27	YNL230C ELA1 SGDID:S00000517	YNL230C	protein catabolic prosess	TAGCAGTAA	UAG	CAGTAA	nd	
28	YOR249C APC5 SGDID:S00000577	YOR249C	cell cycle	TGACAATAA	UGA	CAATAA	nd	
29	YOR255W OSW1 SGDID:S00000578	YOR255W	ascospore formation	TAACAAAAA	UAA	CAAAAA	nd	
30	YOR360C PDE2 SGDID:S00000588	YOR360C	cAMP-mediated signaling	TAGCAAGAA	UAG	CAAGAA	**9.7**	Beznoskova et al. 2015

Figure S10: The list of all *S. cerevisiae* genes bearing a stop codon followed by the CARNNA nucleotide sequence indicating their biological roles. database with confirmed readthrough induction potential in all three cases tested. We created a script to search the 5887 genes from the SGD database for genes with the CARNNA nucleotide context following the stop codon. The table shows the original DNA sequence focused on the stop codon context (original stop plus the following 6 nucleotides). All tested contexts, indicated in the table, do induce readthrough at UGA in comparison to the control SUP45 context (ATAAAT). The complete function of these genes extracted from SGD is given in the Supplementary Excel File.

SUPPLEMENTARY TABLES

Table S1. Yeast strains used in this study.

Strain	Genotype	Source or reference
PBH140[a]	*MATa ade1-14 trp1-289 his3-Δ200 leu2-3,112 ura3-52 tif35Δ* (YEp-TIF35-U)	(1)
PBH156 [a]	*MATa ade1-14 trp1-289 his3-Δ200 leu2-3,112 ura3-52 tif35Δ* (YCp22-g/TIF35-screen)	This study
BY4741/PBH308[b]	*MATa his3Δ1 leu2Δ0 ura3Δ0 met15Δ0*	Euroscarf
PBH267[b]	*MATa his3Δ1 leu2Δ0 ura3Δ0 met15Δ0 pus1Δ*	Euroscarf
PBH303[b]	*MATa his3Δ1 leu2Δ0 ura3Δ0 met15Δ0 pus7Δ*	Euroscarf
PBH270[b]	*MATa his3Δ1 leu2Δ0 ura3Δ0 met15Δ0 trm7Δ*	Euroscarf
PBH309[b]	*MATa his3Δ1 leu2Δ0 ura3Δ0 met15Δ0 mod5Δ*	Euroscarf
H2879	*MATa leu2-3 leu2-112 ura3-52 PRT1*	(5)

[a,b] indicate isogenic strain background

Table S2. Plasmids used in this study.

Plasmid	Description	Source of reference
YCp22-g/TIF35-screen	single copy wt *TIF35-His* in *TRP1* plasmid from YCplac22	(6)
YEp-R/T-UGAC-L	high copy PGK-Renilla-Firefly R/T cassette (stop codon of Renilla is UGA-C; for read-through measurements) in *LEU2* plasmid from YEplac181	(2)
YEp-R/T-CAAC-L	high copy PGK-Renilla-Firefly R/T cassette (stop codon of Renilla is replaced with CAA-C [coding triplet]; for control read-through measurements) in *LEU2* plasmid from YEplac181	(2)
PBB75	high copy PGK-Renilla-Firefly R/T cassette (stop codon of Renilla is UGA-A; for read-through measurements) in *LEU2* plasmid from YEplac181	(1)
PBB76	high copy PGK-Renilla-Firefly R/T cassette (stop codon of Renilla is UGA-G; for read-through measurements) in *LEU2* plasmid from YEplac181	(1)
PBB77	high copy PGK-Renilla-Firefly R/T cassette (stop codon of Renilla is UGA-U; for read-through measurements) in *LEU2* plasmid from YEplac181	(1)
pTH460	high copy PGK-Renilla-Firefly R/T cassette (stop codon of Renilla is CAA-C; for read-through measurements) in *URA3* plasmid from YEplac195	(7)
pTH461	high copy PGK-Renilla-Firefly R/T cassette (stop codon of Renilla is UAA-C; for read-through measurements) in *URA3* plasmid from YEplac195	(7)
pTH469	high copy PGK-Renilla-Firefly R/T cassette (stop codon of Renilla is UAG-C; for read-through measurements) in *URA3* plasmid from YEplac195	(7)
pDB689	high copy PGK-Renilla-Firefly R/T cassette (stop codon of Renilla is UAA-A; for read-through measurements) in *URA3* plasmid from YEplac195	D. Bedwell

pDB725	high copy PGK-Renilla-Firefly R/T cassette (stop codon of Renilla is UAA-G; for read-through measurements) in *URA3* plasmid from YEplac195	D. Bedwell
pDB727	high copy PGK-Renilla-Firefly R/T cassette (stop codon of Renilla is UAA-U; for read-through measurements) in *URA3* plasmid from YEplac195	D. Bedwell
pDB730	high copy PGK-Renilla-Firefly R/T cassette (stop codon of Renilla is UAG-A; for read-through measurements) in *URA3* plasmid from YEplac195	D. Bedwell
pDB731	high copy PGK-Renilla-Firefly R/T cassette (stop codon of Renilla is UAG-G; for read-through measurements) in *URA3* plasmid from YEplac195	D. Bedwell
pDB718	high copy PGK-Renilla-Firefly R/T cassette (stop codon of Renilla is UAG-U; for read-through measurements) in *URA3* plasmid from YEplac195	D. Bedwell
YEp-R/T-UAAC-L	high copy PGK-Renilla-Firefly R/T cassette (stop codon of Renilla is UAA-C; for read-through measurements) in *LEU2* plasmid from YEplac181	(1)
YEp-R/T-UAGC-L	high copy PGK-Renilla-Firefly R/T cassette (stop codon of Renilla is UAG-C; for read-through measurements) in *LEU2* plasmid from YEplac181	(1)
PBB157	high copy PGK-Renilla-Firefly R/T cassette (stop codon of Renilla is UAA-A; for read-through measurements) in *LEU2* plasmid from YEplac181	This study
PBB158	high copy PGK-Renilla-Firefly R/T cassette (stop codon of Renilla is UAG-U; for read-through measurements) in *LEU2* plasmid from YEplac181	This study
PBB159	high copy PGK-Renilla-Firefly R/T cassette (stop codon of Renilla is UAG-A; for read-through measurements) in *LEU2* plasmid from YEplac181	This study

PBB160	high copy PGK-Renilla-Firefly R/T cassette (stop codon of Renilla is UAG-G; for read-through measurements) in *LEU2* plasmid from YEplac181	This study
PBB161	high copy PGK-Renilla-Firefly R/T cassette (stop codon of Renilla is UAA-G; for read-through measurements) in *LEU2* plasmid from YEplac181	This study
PBB162	high copy PGK-Renilla-Firefly R/T cassette (stop codon of Renilla is UAA-U; for read-through measurements) in *LEU2* plasmid from YEplac181	This study
YEplac195	high copy cloning vector, *URA3*	(8)
pTH335	high copy *URA3* vector (pRS426) containing genomic DNA surrounding the *tW(CCA)G1* gene	(1)
PBB97	high copy *tC(GCA)P1* in *URA3* plasmid from pRS426	(1)
PBB90	high copy *URA3* vector (pRS426) containg the *tY(GUA)J2* wild-type gene (=*SUP4*)	(1)
PBB146	high copy *tE(UUC)B* in *URA3* plasmid from pRS426	This study
PBB147	high copy *tK(UUU)D* in *URA3* plasmid from pRS426	This study
PBB148	high copy *tQ(UUG)B* in *URA3* plasmid from pRS426	This study
PBB149	high copy *tE(CUC)D* in *URA3* plasmid from pRS426	This study
PBB150	high copy *tQ(CUG)M* in *URA3* plasmid from pRS426	This study
PBB156	high copy *tK(CUU)P* in *URA3* plasmid from pRS426	This study
PBB134	high copy *tQ(UUG)L* in *URA3* plasmid from pRS426	This study
B998/pRS425	high copy cloning vector, *LEU2*	(9)
B999/pSZ61	high copy *tQ(UUG)L* in *LEU2* plasmid from pRS425	(9)
B1000/pSZ62	high copy *tE(UUC)* in *LEU2* plasmid from pRS425	(9)
B1001/pSZ63	high copy *tK(UUU)* in *LEU2* plasmid from pRS425	(9)
ZPB30	high copy *tC(GCA)P1, tY(GUA)J2* in *URA3* plasmid from pRS426	This study
ZPB31	high copy *tQ(CUG)M, tY(GUA)J2* in *URA3* plasmid from pRS426	This study

ZPB32	high copy *tW(CCA)G1, tY(GUA)J2* in *URA3* plasmid from pRS426	This study
ZPB33	high copy *tW(CCA)G1, tC(GCA)P1* in *URA3* plasmid from pRS426	This study
ZPB34	high copy *tC(GCA)P1, tQ(CUG)M* in *URA3* plasmid from pRS426	This study
ZPB35	high copy *tW(CCA)G1, tQ(CUG)M* in *URA3* plasmid from pRS426	This study
B1002	high copy *tK(CUU)C* in *LEU2* plasmid from pRS425	S.Leidel
B1003	high copy *tQ(CUG)M* in *LEU2* plasmid from pRS425	S.Leidel
B1004	high copy *tE(CUC)D* in *LEU2* plasmid from pRS425	S.Leidel
B1005	high copy *tW(CCA)G1* in *LEU2* plasmid from pRS425	S.Leidel
B1006	high copy *tC(GCA)G* in *LEU2* plasmid from pRS425	S.Leidel
B1007	high copy *tY(GUA)F2* in *LEU2* plasmid from pRS425	S.Leidel
B1008	high copy *tR(UCU)K* in *LEU2* plasmid from pRS425	S.Leidel
B1009	high copy *tG(UCC)G* in *LEU2* plasmid from pRS425	S.Leidel
PBB80	high copy PGK-Renilla-Firefly R/T cassette (stop codon of Renilla is UGA-CAACUA; for read-through measurements) in *URA3* plasmid from YEplac195	(1)
PBB85	high copy PGK-Renilla-Firefly R/T cassette (stop codon of Renilla is UGA-AUAAAU; for read-through measurements) in *URA3* plasmid from YEplac195	(1)
PBB81	high copy PGK-Renilla-Firefly R/T cassette (stop codon of Renilla is UGA-CAGACA; for read-through measurements) in *URA3* plasmid from YEplac195	This study

Table S3. Primers used in this study.

Primer name	Primer sequence (5´to 3´)
PBRFNotI	CTCGAAGCGGCCGCTCTAGAATTACAC
PB95	AATAAGGATCCAAAGCCGTACAGGCGAACGTATATAATT AAAATTC
PB106	CTAGTGGATCCTATAAAAAGAACATATTCATAC
PB130	AATAACTCGAGTTAAACTTGACATAGAAAATCTTTAAGG
PB131	AATAAGGATCCTATCATAATGCAACATTCAAGAAAAATA ATGG
PB132	AATAACTCGAGCTTTAAAATAACATGGACAAATTTATGT CCAGG
PB133	AATAAGGATCCGATACCCTTTTAAAGGTACCGGTTAAAA ATTGAG
PB134	AATAACTCGAGTTTTCAAACCACTCAATTTAAAAAATTGT CAG
PB135	AATAAGGATCCATCTCCATTCTAAGAGTGTCCGATAATT CATG
PB136	AATAACTCGAGTCATTACATAATGAACGTTTCTTTTAGG
PB137	AATAAGGATCCTAACGGTAAATGAATCAGCATGTGAATT TTTCTAG
PB138	AATAACTCGAGTATTTATATTTTTAAGTCACTTATTAAAC G
PB139	AATAAGGATCCCGCTTGCGGCACATGTAATGTAGTTTCT TTC
PB140	AATAACTCGAGAGGTTCCATAAAACCGGAAGTTTTAGTG
PB141	AATAAGGATCCAAAAAAAAAAATGATGGTTTAAATTTCG TAAAATACG
PB142	AATAACTCGAGCCAAATATTCGTTAGATAAAGATGTTTC GG
PB143	AATAAGGATCCCTTGGCAGCGGTTAAGGG
PB222	AATAAGAGCTCATTTTTTACATTTGTTCTATCAG
PB223	AATAAGAGCTCTTGCGTGGATAAGTGTTATTATTCTATT GCC
PB224	AATAAGAGCTCAGGTTCCATAAAACCGGAAGTTTTAGTG
tQ(UUG)	TACCCGGATTCGAACCGGGG
tE(UUC)	TCCGATACGGGGAGTCGAAC
tK(UUU)	TCCTCATAGGGGGCTCGAAC
tQ(CUG)	GTCCCACCCGGATTCGAACT
tE(CUC)	CTCCGAAGCGGGGAGTCGAA
tK(CUU)	GGGGCTCGAACCCCTAACCT
tQ(UUG)L	GTCCTACCCGGATTCGAACC
tQ(UUG)B	GTCTTACCCGGATTCGAACC
5.8S rRNA	GCTGCGTTCTTCATCGATGCGAGAACCAA
5S rRNA forward	GTTTCCCGTCCGATCAACTGTAGTTAAGC
5S rRNA reverse	GATTGCAGCACCTGAGTTTCGCG
RPL41A forward	CGAAATGAGAGCCAAGTGG
RPL41A reverse	ATGCAATTTAGATCCATTATGAGG
Rluc forward	TTTTGTCGGCCATGATTGGG
Rluc reverse	CAGGCCATTCATCCCATGATTC

Fluc forward	TTTTGGAGCACGGAAAGACG
Fluc reverse	ACCTTTCGGTACTTCGTCCAC
TIF32	CAAATGTCGACGTGCGATTGACAGACAGGATCCTTCAA CTTCCCTGAGCTCG
